# The longevity-associated variant of BPIFB4 improves a CXCR4-mediated striatum–microglia crosstalk preventing disease progression in a mouse model of Huntington’s disease

**DOI:** 10.1038/s41419-020-02754-w

**Published:** 2020-07-18

**Authors:** Alba Di Pardo, Elena Ciaglia, Monica Cattaneo, Anna Maciag, Francesco Montella, Valentina Lopardo, Anna Ferrario, Francesco Villa, Michele Madonna, Enrico Amico, Albino Carrizzo, Antonio Damato, Giuseppe Pepe, Federico Marracino, Alberto Auricchio, Carmine Vecchione, Vittorio Maglione, Annibale A. Puca

**Affiliations:** 1 IRCCS Neuromed, 86077 Pozzilli, Italy; 2 Department of Medicine, Surgery and Dentistry “Scuola Medica Salernitana”, University of Salerno, 84081 Baronissi, Italy; 3 Cardiovascular Research Unit, IRCCS MultiMedica, 20138 Milan, Italy; 4 TIGEM (Telethon Institute of Genetics and Medicine), 80078 Pozzuoli, Italy; 5 Department of Translational Medicine, “Federico II” University, Naples, Italy; 6 Present Address: Neurodevelopmental Behavior Core, F.M. Kirby Neurobiology Center, Boston Children’s Hospital, Harvard Medical School, Boston, MA USA

**Keywords:** Huntington's disease, Huntington's disease

## Abstract

The longevity-associated variant (LAV) of the bactericidal/permeability-increasing fold-containing family B member 4 (BPIFB4) has been found significantly enriched in long-living individuals. Neuroinflammation is a key player in Huntington’s disease (HD), a neurodegenerative disorder caused by neural death due to expanded CAG repeats encoding a long polyglutamine tract in the huntingtin protein (Htt). Herein, we showed that striatal-derived cell lines with expanded Htt (STHdh Q^111/111^) expressed and secreted lower levels of BPIFB4, when compared with Htt expressing cells (STHdh Q^7/7^), which correlated with a defective stress response to proteasome inhibition. Overexpression of LAV-BPIFB4 in STHdh Q^111/111^ cells was able to rescue both the BPIFB4 secretory profile and the proliferative/survival response. According to a well-established immunomodulatory role of LAV-BPIFB4, conditioned media from LAV-BPIFB4-overexpressing STHdh Q111^/111^ cells were able to educate Immortalized Human Microglia—SV40 microglial cells. While STHdh Q^111/111^ dying cells were ineffective to induce a CD163 + IL-10^high^ pro-resolving microglia compared to normal STHdh Q^7/7^, LAV-BPIFB4 transduction promptly restored the central immune control through a mechanism involving the stromal cell-derived factor-1. In line with the in vitro results, adeno-associated viral-mediated administration of LAV-BPIFB4 exerted a CXCR4-dependent neuroprotective action in vivo in the R6/2 HD mouse model by preventing important hallmarks of the disease including motor dysfunction, body weight loss, and mutant huntingtin protein aggregation. In this view, LAV-BPIFB4, due to its pleiotropic ability in both immune compartment and cellular homeostasis, may represent a candidate for developing new treatment for HD.

## Introduction

Huntington’s disease (HD) is an autosomal dominant inherited neurodegenerative disorder. The disease-causing mutation is a CAG repeat expansion within the HTT gene encoding huntingtin protein (Htt) that when mutated (mHtt) exerts a variety of undesirable toxic effects in both neuronal and nonneuronal cells. Specifically, CAG repeat length of up to 35 repeats generally does not result in the onset of HD^1,2^. However, individuals with repeat numbers from 36 up should be viewed at as at risk of developing HD^3^. CAG repeat lengths of >40 are associated with a definite onset of HD within a normal lifespan^1^.

A diffuse and gradual degeneration of the striatum and cortex represents the main hallmark of HD^4^ which is characterized by brain atrophy, cognitive decline, behavioral disturbances, body weight loss, and movement disorders^5^. Specifically, every HD patient has a specific pattern of motor dysfunction: mainly uncontrolled choreic movements but slowness both in starting movement (akinesis) and in executing a movement (bradykinesia) and decrease in all motor activities (hypokinesia) are also presented by HD patients^6^.

Although there are several approved therapies to help manage many of these symptoms and to maintain patients’ quality of life for as long as possible, no cure is currently available for the disease.

An investigative immunomodulatory drug called laquinimod tested in a clinical trial (LEGATO-HD, clinicaltrials.gov ID: NCT02215616) has resulted in encouraging improvement of motor and behavioral disabilities^7^ even though it failed to meet the primary endpoint of the trial^8^. The rationale behind the use of laquinimod was its efficiency in reducing inflammation, a putative underlying mechanism that causes the death of nerve cells in HD. Following the same strategy, new therapeutic approaches targeting striatum-immune cells crosstalk in brain are warranted.

Actual knowledge of neuroinflammation in HD underpins a crucial role of microglia in disease progression. These brain resident immune cells of myeloid origins oscillate between a surveilling and activated state to ensure the brain homeostasis. Microglia demonstrate a dynamic, context-dependent expression phenotype determined by local environment and recent exposure to stimuli. In inflammatory process of the central nervous system (CNS), activated microglia can produce pro-inflammatory cytokines and mediators such as interleukin (IL) 1β, IL-6, tumor necrosis factor-α, CC-chemokine ligand 2, nitric oxide, and reactive oxygen species which contribute to dysfunction of neural network and promoting inflammatory reaction. On the other hand, a pro-resolving state of microglia can produce anti-inflammatory cytokine, mainly IL-10, and expresses several receptors and neurotrophic factors that are implicated in inhibiting inflammation and restoring brain homeostasis (e.g., neurogenesis, trophism, motility, synaptic plasticity, survival, etc.). Even though the previous dichotomy can resemble to M1 and M2 polarization states of peripheral blood monocyte-derived macrophages, microglia display a more complex phenotype due to its extreme heterogeneity in transcriptomic and proteomic profiles, localization, patterns of response to different stimuli and gender and age dependence^9^. Regardless of the semantic question, it has been proposed that an imbalance between pro- and anti-inflammatory state of microglia might contribute to bipolar disorder, amyotrophic lateral sclerosis, multiple sclerosis (MS), and Rett syndrome^10–13^.

Of note, depending on the disease stage, a M1/M2 microglia imbalance has been reported to be either detrimental or beneficial also in a number of human and animal HD studies^14^. Indeed changes in microglia, astrocytes, circulating cytokine levels, infiltration of macrophages along with changes in the transcription of genes associated with the control of inflammation have been identified in HD^15^.

The bactericidal/permeability-increasing fold-containing family B member 4 (BPIFB4) is a member belonging to the BPI/lipopolysaccharide-binding protein family of antibacterial components that participates in host protection through antimicrobial, surfactant, and immunomodulatory properties^16,17^. Of note, circulating BPIFB4 levels are constitutively increased in healthy long-living individuals (LLIs) as compared to frail ones and young controls^18,19^. Moreover, carriers of the longevity-associated variant (LAV) of BPIFB4 associate with exceptional longevity^20^ and display exclusive beneficial effects in many molecular pathways.

First, BPIFB4 has been shown to preserve cellular homeostasis through the activation of many heat shock protein (HSP) family members involved in maintaining and restoring favorable proteostatis^20,21^, which is progressively lost during aging and in neurodegenerative disease. Indeed LAV-BPIFB4 associates with reduced frailty in humans and its transfer prevents frailty progression in old mice^22^.

Furthermore, gene therapy employing LAV-BPIFB4 resulted in improved revascularization and endothelial function^20^, mainly through the maintenance of nitric oxide bioavailability. Concerning its effects on immune compartment, we also found that LAV-BPIFB4 redirects peripheral monocyte polarization toward regulatory IL-10- and TGF-beta 1-producing dendritic cells in human healthy donor^17^. More recently, LAV-BPIFB4 has been demonstrated to blunt atherosclerosis in an in vivo mouse and in ex vivo human model^23^. Specifically, we observed that in atherosclerosis-prone ApoE knockout animals, LAV-BPIFB4 gene transfer causes a CXCR4-dependent redistribution of the two major monocyte subsets with increased circulating Ly6C^high^ cells and reduced Ly6C^low^ subsets, a protective macrophage polarization toward the pro-resolving M2 phenotype, and a reduction in T-cell activation^23^. Similarly, in human atherosclerotic monocytes, LAV-BPIFB4 exerted a macrophage M2-polarizing effect via CXCR4-dependent mechanism^23^.

In this view, the LAV-BPIFB4 pleiotropic ability, both in immune compartment and cellular homeostasis, challenged us to investigate its potential role as a new promising immunoregulatory agent in HD.

In the present study, we shed light on the LAV-BPIFB4 therapeutic potential by gene transfer adopting either a cellular model of striatal-derived cell lines and their crosstalk with microglia or an animal model (R6/2 mice) of HD^24^.

## Results

### BPIFB4 is expressed at different levels in STH*dh* striatal cells model of HD

**Fig. 1:**
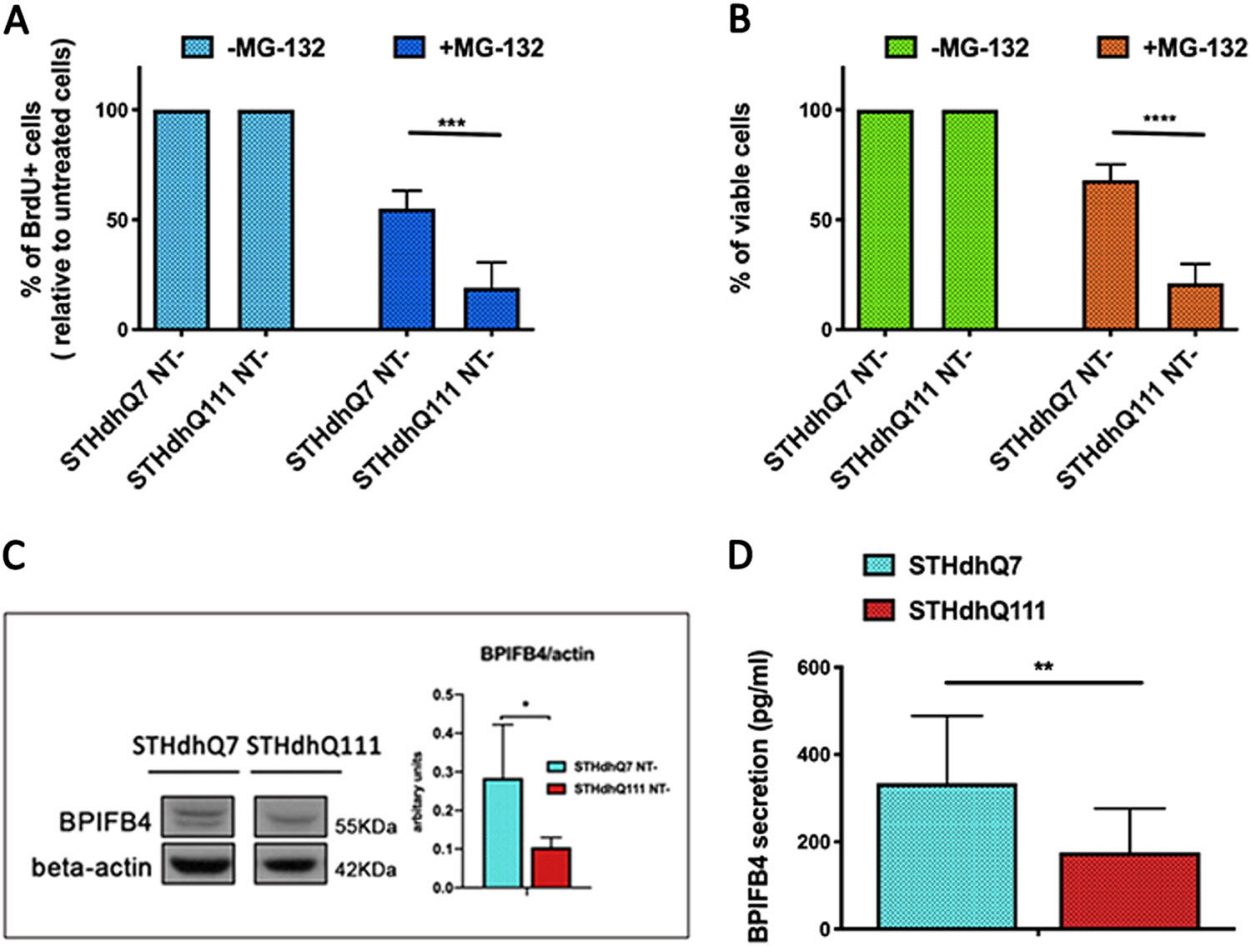
Different features in STHdh Q^7/7^ and STHdh Q^111/111^ cell line concerning the sensitivity to stress induced by proteasome blocking and the BPIFB4 expression profile. **a**, **b** STHdh Q^7/7^ and STHdh Q^111/111^ were treated with MG-132 (5 µM) for 24 h. After cells were assayed for the BrdU incorporation (**a**) and cell viability (**b**). Panel **a** shows the percentage of BrdU+ proliferating cells after treatment using a BrdU-ELISA assay. Panel **b** shows the percentage of viable cells after treatment using a Cell Counting Kit-8 (CCK-8) colorimetric assays. For both assays, the effect of the treatment was compared in the two cell lines independently. **c** Representative blot of BPIFB4 expression level and results of densitometric analysis of the protein in STHdh Q^7/7^ and STHdh Q^111/111^ cell line. Beta-actin served as loading controls (**c**). Panel **d** shows the different levels of protein secretion in STHdh Q^7/7^ and STHdh Q^111/111^ cell line. All experiments were performed in three independent biological experiments and for three technical replicates per sample. Pairwise comparisons statistically significant are indicated (ANOVA; *P < 0.05, **P < 0.01, ***P < 0.001, ****P < 0.0001).

STH*dh* striatal cells represent a widely used cell culture model for studying cellular aspects of HD pathology. In the present study, specifically, we used STH*dh* Q^7/7^ and STH*dh* Q^111/111^ cell lines (which we refer to as Q7 and Q111, respectively, in figures) derived from a knock-in mouse model of HD and endogenously expressing full-length Htt with either short (Q7) or long (Q111) polyglutamine (polyQ) repeats^25^. A putative mechanism for toxicity of long polyQ proteins is through a dysregulated proteasome activity. This is a multicatalytic protein complex which controls cellular protein turnover and which virtually governs almost all basic cellular processes, including the cell cycle, signal transduction, and survival^26^. Thus, to corroborate the adequacy of the cell model used to describe our results, we started to test the response to proteasome blocking in term both of cell cycle arrest (Fig. 1a) and cytotoxicity (Fig. 1b) on STH*dh* Q7/Q7 and STH*dh* Q111/Q111 cell lines treated or not with the proteasome inhibitor MG-132 (5 μM). As reported in Fig. 1a, after 24-h treatment STH*dh* Q7/Q7 had a higher percentage of BrdU+ proliferating cells (*P < 0.001) compared to STH*dh* Q111/Q111 cells. The observed differences in proliferative rate were in line with the reduced amount of STH*dh* Q111/Q111 viable cells (*P < 0.001) compared to STH*dh* Q7/Q7 cells, explainable with a better ability of the latter to cope with the conditions of proteotoxic stress (Fig. 1b). The altered response of two cell lines led us to explore the differential protein level of BPIFB4. Western blot analysis showed a significant downregulation of the BPIFB4 immunoreactivity (*P < 0.05) in mutant STH*dh* Q111/Q111 cells compared to wild-type STH*dh* Q7/Q7 (Fig. 1c, right histogram). In agreement, enzyme-linked immunosorbent assay (ELISA) analysis showed a decreased BPIFB4 secretion in the STHdh Q111/Q111 compared to the nonmutant striatal cells (Fig. 1d; *P < 0.01).

### LAV-BPIFB4 overexpression in STH*dh* Q^111/111^ in vitro leads to the recovery of the BPIFB4 secretory profile and blunts the cytotoxic and cytostatic responses to proteasome blocking

**Fig. 2:**
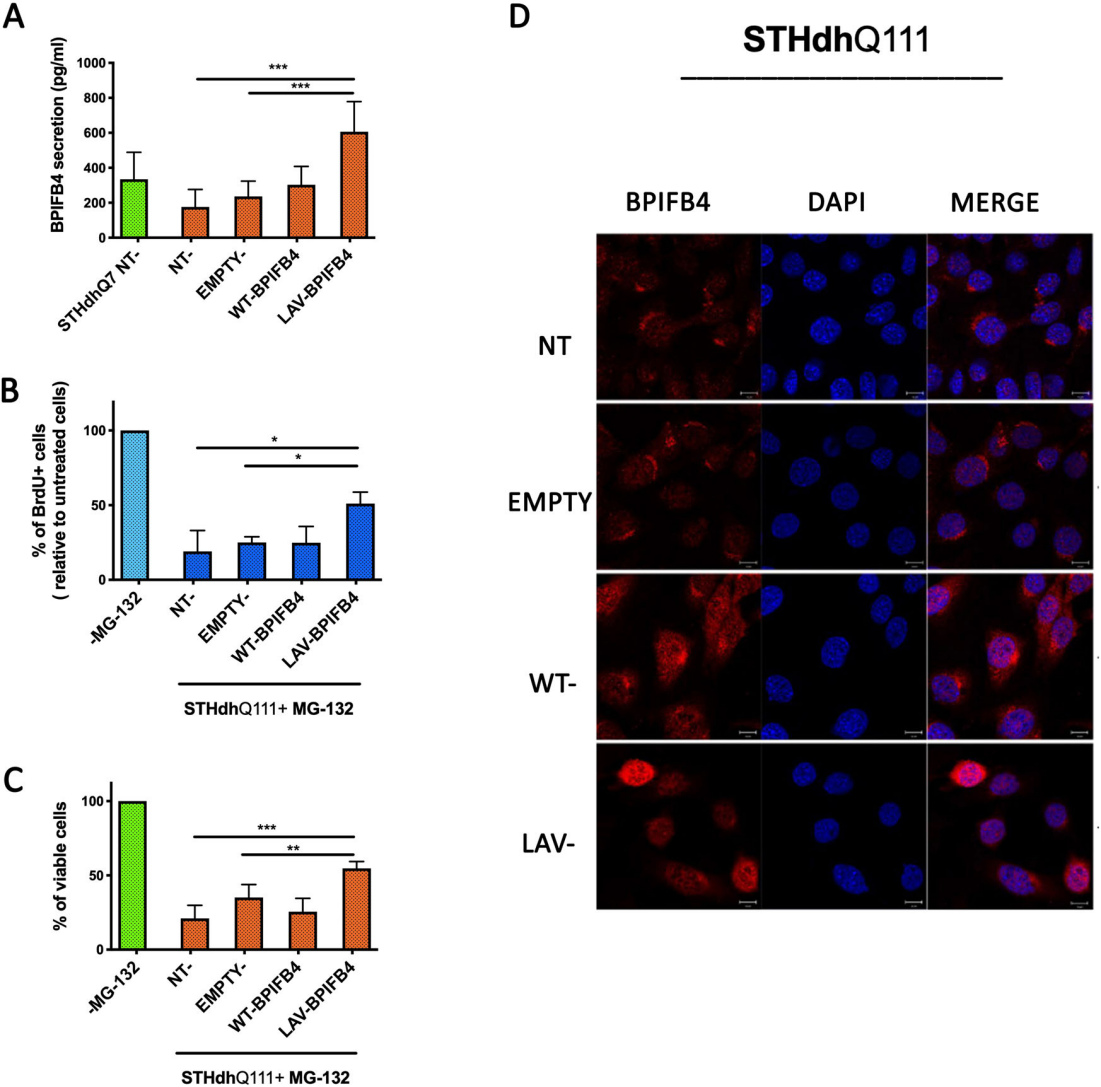
Effects of BPIFB4 infection on the defective proliferation and the viability and the BPIFB4 protein secretion in STHdh Q^111/111^ cell line. STHdh Q^111/111^ cells were infected with empty lentiviral vector or particles encoding either WT- or LAV-BPIFB4 as described in the “Materials and Methods” section. Histograms report the levels of BPIFB4 protein secretion quantified by ELISA assay (**a**), the percentage of BrdU+ proliferating (**b**), and viable infected STHdh Q^111/111^ cells (**c**) after 24-h treatment with MG-132. One week after infection, the levels of BPIFB4 (red) in STHdh Q^111/111^ and in normal untreated NT STHdh Q^7/7^ for comparison were analyzed by immunofluorescence staining. Nuclei were stained with DAPI (**d**). All experiments were performed in three independent biological experiments and for three technical replicates per sample. Pairwise comparisons statistically significant are indicated (ANOVA; *P < 0.05, **P < 0.01, ***P < 0.001).

Because BPIFB4 cellular levels were significantly reduced in STH*dh* Q^111/111^, we sought to prove the ability of the overexpression of human BPIFB4 isoforms to rescue the defective response in STH*dh* Q^111/111^. To this end, we examined the secretory profile and changes both in the proliferation and the cell viability of the mutant STH*dh* Q^111/111^ in response to lentivirus-mediated overexpression of human BPIFB4 isoforms (both WT- and LAV-BPIFB4). At functional level, a recovery of the BPIFB4 secretory profile (Fig. 2a) and a better response to the inhibition of the proteasome (Fig. 2b, c) were observed following infection with LAV-BPIFB4. Indeed, the percentage of STH*dh* Q^111/111^ viable and proliferating cells under MG-132-induced stress was statistically higher in striatal cells infected with LAV-BPIFB4 than either untreated or empty vector-infected striatal cells (*P < 0.05 in BrdU incorporation assay in Fig. 2b and, respectively, *P < 0.001 and *P < 0.01 in panel Fig. 2c) (Fig. 2b, c). Most importantly, the restoration of a defective proliferation (Fig. 2b) and the survival recovery (Fig. 2c) paired with the statistically significant increase in the BPIFB4 secretory profile that shows the highest BPIFB4 protein levels by STHdh Q^111/111^ infected with LAV-BPIFB4 (Fig. 2a; *P < 0.001).

The achieved results supported the protective effects of LAV-BPIFB4 and BPIFB4 secretion against insults induced by mHtt in STHdh Q^111/111^, such as impaired heat shock response and loss of proteostasis described in previous reports^27^, processes already showed to be activated by BPIFB4 overexpression^20^.

Of note, immunofluorescence staining using anti-BPIFB4 antibody revealed not only an expected overexpression of the protein in infected SSTHdh Q^111/111^ cells, but also a different BPIFB4 localization (red staining) within the cell and the nucleus (blue-DAPI-staining). Specifically, a main nuclear distribution of BPIFB4 was acquired in response to LAV-BPIFB4 infection in STHdh Q111^/111^ (Fig. 2d). At the contrary, BPIFB4 was more restricted to the perinuclear region following overexpression of WT-BPIFB4 isoform (Fig. 2d). The different observed dynamics between WT- and LAV-BPIFB4 could be associated with the different pro-survival response by the two isoforms.

### LAV-BPIFB4-infected STH*dh* Q^111/111^ displays an anti-inflammatory and pro-resolving M2-polarizing effect on Immortalized Human Microglia—SV40 (HM-SV40) microglial cells

**Fig. 3:**
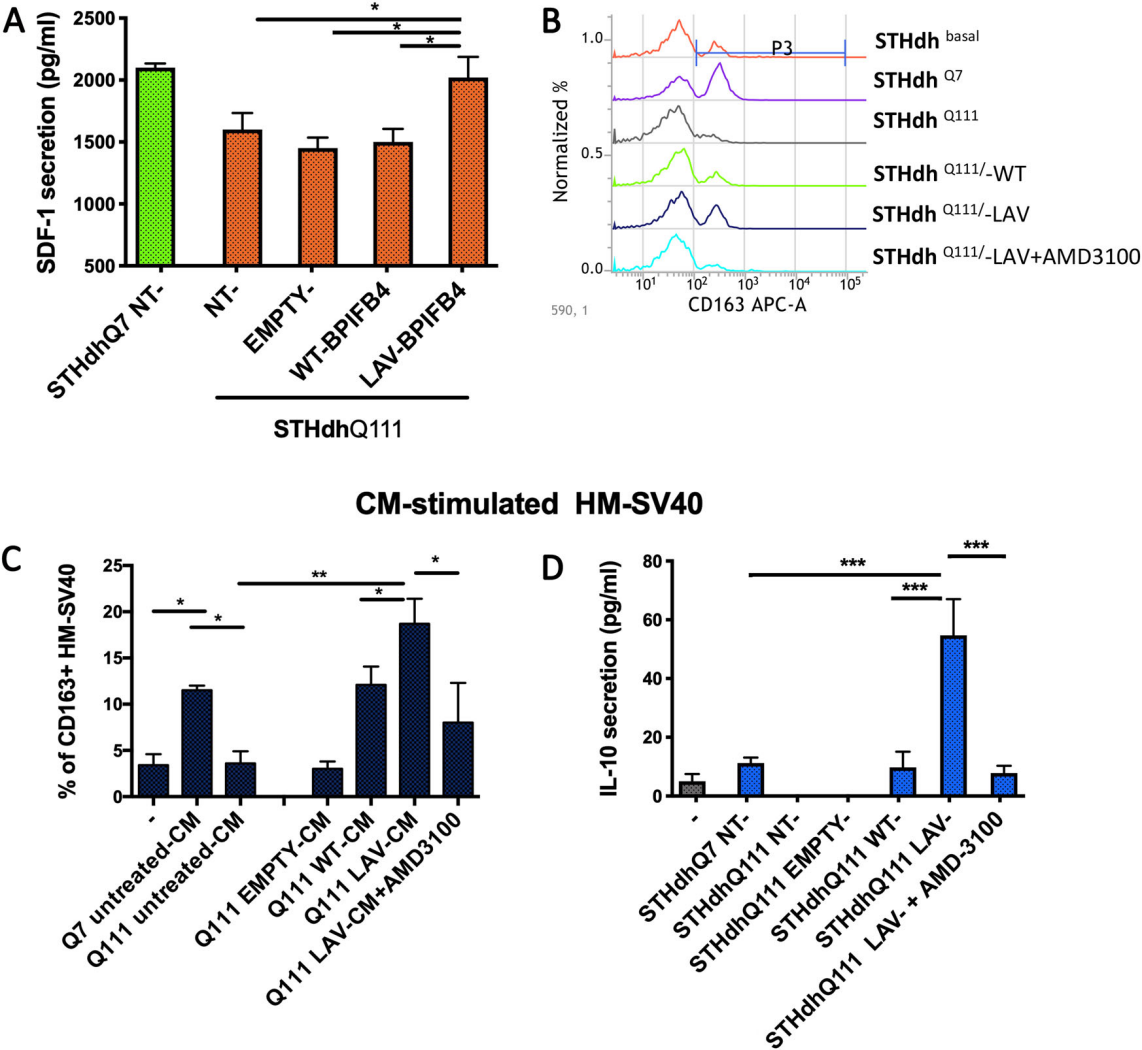
Analysis of anti-inflammatory and M2-polarizing action of CM from LAV-BPIFB4-infected striatal cells. **a** ELISA quantification of SDF-1 levels in cell culture medium from both BPIFB4-infected STHdh Q^111/111^ and STHdh Q^7/7^ for comparison. HM-SV40 were cultured in vitro in complete medium with 20% CM from both BPIFB4-infected STHdh Q^111/111^ and STHdh Q^7/7^, for comparison. Where indicated, the HM-SV40 were pretreated with CXCR4 inhibitor AMD3100 (20 µM). The panel **b** shows FACS histogram profiles of CD163 protein levels at the cell surface of HM-SV40 target cells, after 48 h of treatment. **c** Bars graph in panel reports the percentage ± SD of CD163+ viable HM-SV40 cells under different treatment conditions. **d** IL-10 secretion from treated microglia for 48 h. Cell culture supernatants were collected and cytokines secretion was determined using bead-based multiplex ELISA. All experiments were performed in three independent biological experiments and for two technical replicates per sample. Results were expressed as the mean ± SD of all sample determinations. Pairwise comparisons statistically significant are indicated (ANOVA; *P < 0.05, **P < 0.01, ***P < 0.001).

As recently reported by our group, LAV-BPIFB4-treated mice showed increased stromal cell-derived factor-1 (SDF-1) levels in peripheral blood and myocardium. SDF-1 upregulation was instrumental to LAV-BPIFB4-induced benefit as both haemodynamic and structural improvements were inhibited by an orally active antagonist of the SDF-1, CXCR4 receptor^28^. Beyond its chemotactic activity, SDF-1/CXCR4 axis enhances the immunomodulation of the secreting cells in alleviating the inflammatory processes^29^. Because we previously proved that BPIFB4 has a macrophage M2-polarizing effect via CXCR4-dependent mechanism^23^, we decided to analyze SDF-1 chemokine secretion in our experimental model after BPIFB4 infection. Interestingly, our current ELISA analysis confirmed that STH*dh* Q^111/111^ infected with LAV-, but not WT-BPIFB4, secreted higher level of SDF-1 compared to the untreated striatal cells (Fig. 3a; *P < 0.05). Next, to test the M2-polarizing effects of soluble factors produced by striatal cells after infection, we used STH*dh* medium (CM) to conditionate HM-SV0 microglial cells in vitro. The panels b and c in Fig. 3 showed a cytofluorimetric analysis of CD163 surface marker on HM-SV40 microglia. Interestingly, normal cells (STH*dh* Q^7/7^) induced the cell surface upregulation of CD163 pro-resolving marker on HM-SV40 (*P < 0.05). While this ability was lost (*P < 0.05) in mutant STH*dh* Q^111/111^ cells, where a significant increase in the percentage of CD163+ cells (**P < 0.001) and IL-10 release (***P < 0.0001) (Fig. 3d) was observed after 48-h treatment with CM from LAV-BPIFB4-infected STH*dh* Q^111/111^. The CXCR4-dependent mechanism was confirmed by the decreased CD163 amount (*P < 0.05) and IL-10 secretion (***P < 0.0001) after treating HM-SV40 microglial cells with the CXCR4 antagonist AMD3100 (20 μM).

### LAV-BPIFB4 blunts progression of Huntington’s disease in vivo

**Fig. 4:**
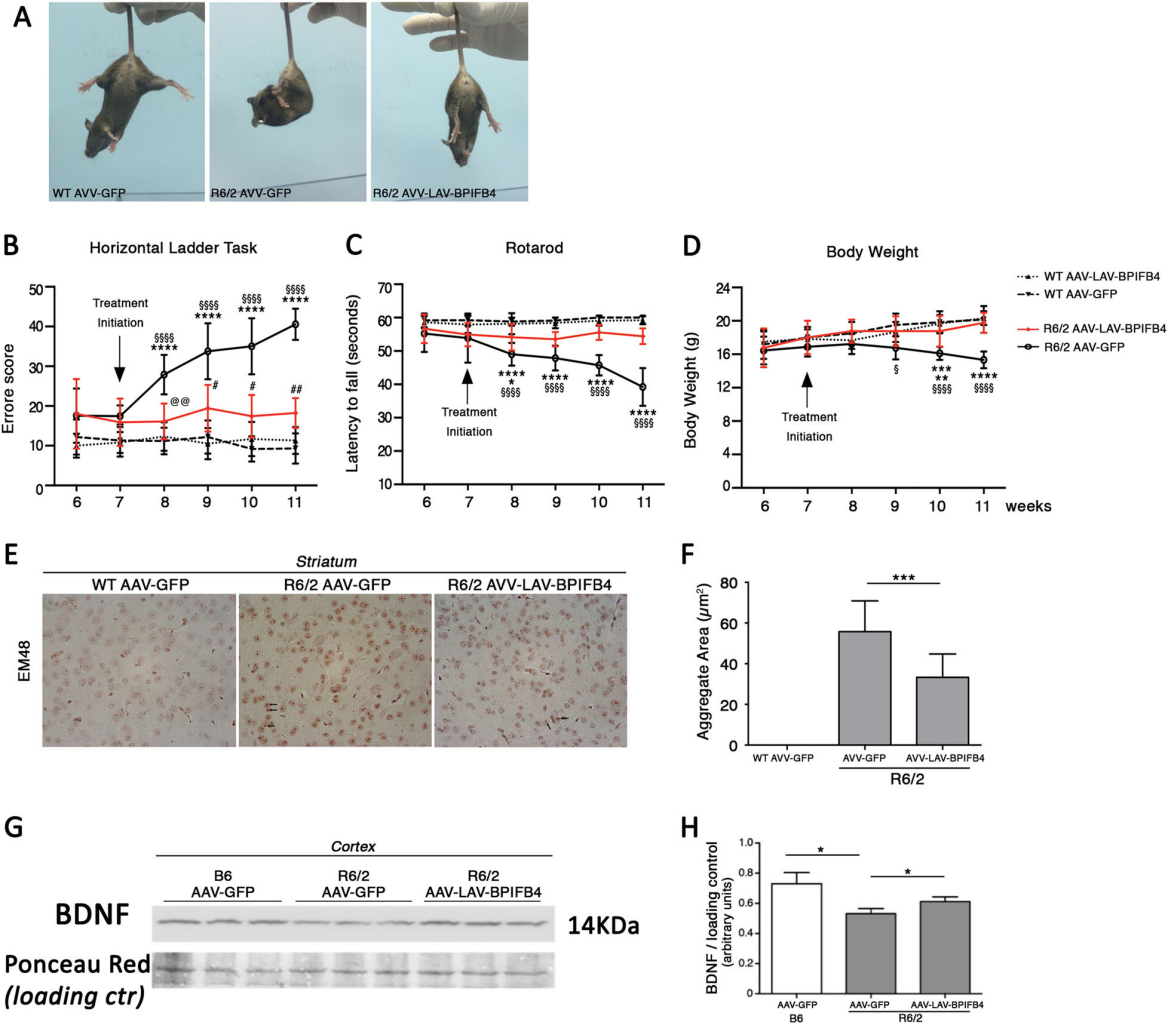
AAV-LAV-BPIFB4 injection stopped disease progression in manifest R6/2 mice by reducing mHtt aggregation in the striatum and restoring normal BDNF levels in the cortex. **a** Mouse clasping phenotype in AAV-GFP-injected B6 mice and AAV-LAV-BPIFB4- and AAV-GFP-injected manifested (11 weeks old) R6/2 HD mice. **b** Horizontal ladder task, **c** rotarod analyses of motor performance in AAV-LAV-BPIFB4- and AAV-GFP-injected manifested R6/2 HD female mice and age- and gender-matched C57BL6 littermates. Each data point represents the average performance ± SD of 6–9 mice for each group of mice. *P < 0.05; ****P < 0.0001 (AAV-LAV-BPIFB4-injected mice versus AAV-GFP-injected mice). ^§§§§^*P* < 0.0001 (AAV-GFP-injected C57BL6 versus AAV-GFP-injected R6/2). ^#^*P* < 0.05; ^##^*P* < 0.001 (AAV-GFP-injected C57BL6 versus AAV-LAV-BPIFB4-injected R6/2). ^@@^*P* < 0.001 (AAV-LAV-BPIFB4-injected C57BL6 versus AAV-LAV-BPIFB4-injected R6/2). **d** Analysis of mouse body weight of all groups of mice. Each data point represents the average of body weight ± SD of 6–9 mice. *P < 0.05; **P < 0.01; ***P < 0.001 (AAV-LAV-BPIFB4-injected mice versus AAV-GFP-injected mice). ^§^*P* < 0.05; ^§§§§^*P* < 0.0001 (AAV-GFP-injected B6 versus AAV-GFP-injected R6/2) (two-way ANOVA with Bonferroni posttest). **e**, **f** Representative micrographs of striatal mutant Htt aggregate revealed by EM48-specific antibody and analysis of their area expressed as µ2 (**f**). Data are expressed as mean ± SD. *N* = 5; ***P < 0.001 (unpaired *t*-test). Representative immunoblotting (**g**) and densitometric analysis (**h**) of BDNF levels in the cortex of AAV-GFP-injected B6 and AAV-GFP-injected and AAV-LAV-BPIFB4-injected R6/2 mice. Data are expressed as mean ± SD. *N* = 5; *P < 0.05 (unpaired *t*-test).

In order to elucidate if the beneficial effect of LAV-BPIFB4 in vitro might be reproducible also in vivo in HD, manifested (7 weeks old) R6/2 female mice and age-matched control littermates were injected into femoral arteries with single dose of 1 × 10^13^ GC/kg of adeno-associated viral (AAV)-GFP, AAV-WT-BPIFB4, or AAV-LAV-BPIFB4 vectors and motor function was then evaluated. Infection was performed at 7 weeks of age and effects were examined for the following 4 weeks. To evaluate efficacy of the potential therapy, paw clasping behavior, a stereotypic phenotype widely used to study disease progression and motor function was analyzed by using horizontal ladder task and rotarod. Changes in the body weight, which is classically associated with disease progression, were also assessed. As reported in Fig. 4, AAV-LAV-BPIFB4 treatment reduced paw clasping behavior (Fig. 4a) and significantly slowed down the progression of motor deficits as assessed by horizontal ladder task and rotarod in R6/2 mice (Fig. 4b, c).

No effects on motor behavior were observed in control mice, nor in R6/2 mice administered with AAV-WT-BPIFB4 (Supplementary Fig. 1). Interestingly, AAV-LAV-BPIFB4 treatment preserved HD mice from the body weight loss, which normally occurs as the disease progresses (Fig. 4d). From the mechanistic point of view, administration of AAV-LAV-BPIFB4 was associated with reduced area of mHtt aggregates in the striatum of HD mice (Fig. 4e, f) and with restoration of normal levels of brain derived neurotrophic factor (BDNF) in the cortex (Fig. 4g, h).

### LAV-BPIFB4 therapeutic effects are blunted by AMD3100

**Fig. 5:**
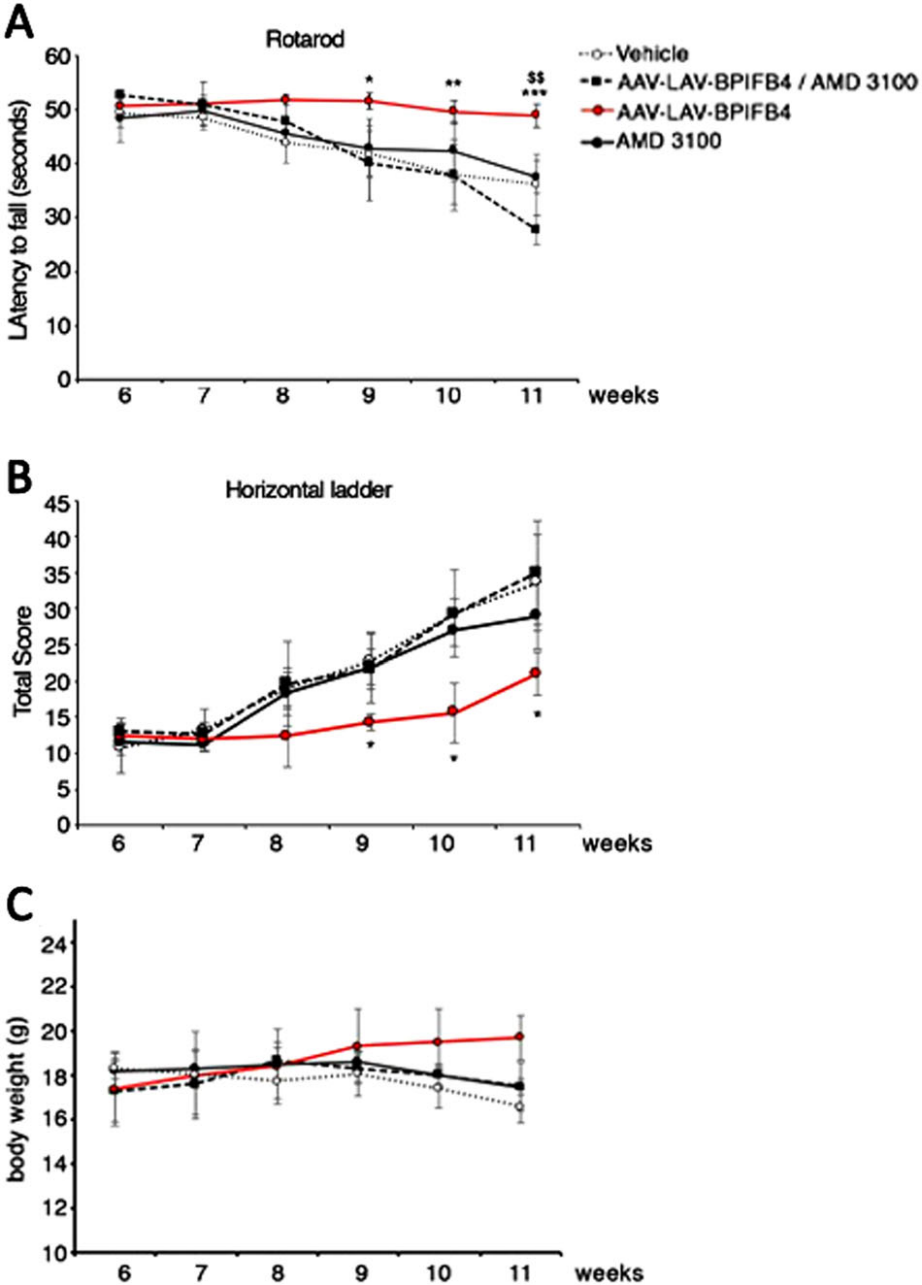
The improvement of motor function engendered by LAV-BPIFB4 in R6/2 mice is blunted by inhibition of CXCR4. **a** Horizontal ladder task and **b** rotarod analyses of motor performance in R6/2 female mice administered AAV-GFP or AAV-LAV-BPIFB4 with or without the CXCR4 inhibitor AMD3100. Each data point represents the average performance ± SD of 3–4 mice per group. Unpaired *t*-test: *P < 0.05; **P < 0.01; ***P < 0.001 (AAV-LAV-BPIFB4-injected mice versus AAV-LAV-BPIFB4-injected mice + AMD3100). **c** Body weight in all groups of mice. Each data point represents the average of body weight ± SD of 3–4 mice.

The beneficial effects on HD progression were blunted when the AAV-LAV-BPIFB4-infected R6/2 female mice were daily treated intraperitoneally with CXCR4 antagonist, AMD3100, at the concentration of 5 mg/kg until the end of treatment. Indeed, motor function tests, horizontal ladder task, and rotarod demonstrated that the retrieval of motor performance in R6/2 mice infected with AAV-LAV-BPIFB4 did not occur in mice treated with AMD3100 (Fig. 5). Similarly as shown, weight loss was more pronounced in R6/2 mice infected with AAV-LAV-BPIFB4 + AMD3100 compared with mice treated with AAV-LAV-BPIFB4 alone, overlapping with untreated mice (Fig. 5).

### LAV-BPIFB4 treatment partially reverts the RNA signature of HD

Aberrant gene expression represents one of the key factors in the pathogenesis of HD^24^. In order to assess any potential effect that LAV-BPIFB4 may have on gene transcription in R6/2 mice, we performed RNAseq analysis on striatum specimens of AAV-GFP-, AAV-WT-BPIFB4-, and AAV-LAV-BPIFB4-injected mice.

Principal component analyses and Euclidean distance analysis were applied to the 13 samples (5 AAV-GFP, 3 AAV-WT-BPIFB4, and 5 AAV-LAV-BPIFB4) to exclude outliers (Supplementary Fig. 2): two AAV-GFP vectors and two AAV-LAV-BPIFB4-injected samples have been excluded before the gene differential expression analyses.

**Fig. 6:**
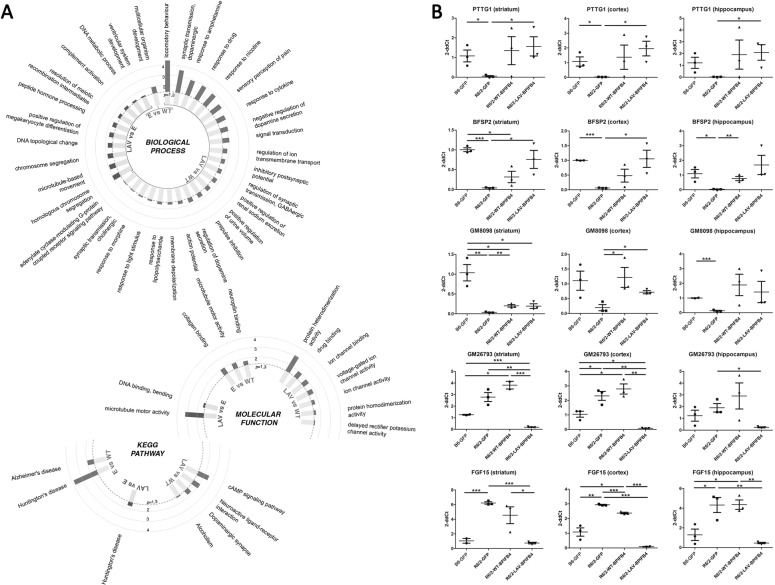
GO and gene expression analysis in striatum, cortex, and hippocampus specimens. **a** Circular histograms represent the results of Gene Ontology analysis on genes differentially expressed with a false discovery rate (FDR) ≤ 0.05 in striatum specimens of AAV-GFP-, AAV-WT-BPIFB4-, and AAV-LAV-BPIFB4-injected R6/2 mice. Biological process, KEGG pathway, and molecular function analyses identified different possible mechanisms modulated by gene therapy. Values are represented as logarithmical *P* values of expression fold change. **b** Graphs show the results of the qPCR validation step on genes showing a fold change higher than 5 in AAV-GFP versus AAV-LAV-BPIFB4 treatment comparison in RNAseq analysis. Total mRNA has been collected from striatum, cortex, and hippocampus specimens. Statistic was performed using two-tailed *t*-test.

Gene Ontology analysis on genes differentially expressed with a false discovery rate ≤0.05 (Supplementary Tables 1–3) showed interesting categories that emerged depending on the comparison analyzed (Fig. 6a and Supplementary Tables 4–6). In biological process analysis, AAV-LAV-BPIFB4 versus AVV-GFP vector comparison showed, among the top categories, microtube-based movement, while AAV-LAV-BPIFB4 versus AAV-WT-BPIFB4 showed locomotor behavior and dopaminergic synaptic transmission. In KEGG pathway analysis the only category that emerged was HD in the comparison between AAV-LAV-BPIFB4- and AAV-GFP-treated mice, while between AAV-LAV- and AAV-WT-BPIFB4 the highest score has been reached by cAMP signaling pathway. The molecular function analysis identified microtubule motor activity category in the comparison of AAV-LAV-BPIFB4-treated animals versus AAV-GFP-treated mice, and protein heterodimerization activity category in the comparison between AAV-LAV- and AAV-WT-BPIFB4 treatments. Overall, these results point to a modulation of categories of genes that are strongly correlated with neurodegenerative disease and specifically of HD.

The expression of the genes showing a fold change higher than 5 in empty versus AAV-LAV-BPIFB4 comparison was further tested by quantitative PCR on RNA from striatum (Table 1), and compared with the expression in striatum from wild-type mice (Fig. 6b). Among the genes upregulated after AAV-LAV-BPIFB4 gene therapy, pituitary tumor-transforming gene 1 (PTTG1) (27.0-fold change increase), beaded filament structural protein 2 (BSFP2) (18.9-fold change increase), and Gm8098 (5.5-fold change increase) confirmed the overexpression in qPCR analysis, while HBA-a2 (1.5-fold change increase) and 9430037G07Rik (1.14-fold change increase) failed the validation. Gm26793, which is downregulated in AAV-LAV-BPIFB4-treated animals, is a long noncoding RNA that resides on fibroblast growth factor 15 (FGF15). Once confirmed its downregulation by qPCR (7.9-fold change decrease), we interrogated FGF15 (by adopting primers that did not overlap with GM26793) and surprisingly, we recorded a similar downregulation (8.3-fold change decrease) indicating that what recorded as GM26793 altered expression reflects FGF15 expression (Fig. 6b and Table 1). Among the downregulated genes, EPS8L1, C1RB, and Gm18190 failed the validation. To be noted, the validation of Gm37460, Gm7335, and Gm13394 through qPCR was not possible because of the impossibility to design functional primers.

**Table 1:** Differential expression (RNAseq and qPCR analyses) in striatum of mice infected with empty vector compared to AAV-LAV-BPIFB4.

Gene	*P* value	FDR	FC (RNAseq)	FC (qPCR)
Hba-a2	6.97E−20	4.40E−16	−29.04	1.47
Pttg1	2.24E−74	2.82E−70	−25.58	−27.03
Gm37460	1.73E−07	0.000256779	−23.92	N/A
9430037G07Rik	0.00012483	0.049276587	−21.22	1.14
Bfsp2	6.26E−10	1.44E−06	−13.80	−18.90
Gm8098	3.42E−12	1.44E−08	−7.67	−5.47
Gm7335	7.05E−23	5.94E−19	8.47	N/A
Eps8l1	9.87E−05	0.041551904	12.28	1.22
Gm26793	3.08E−13	1.56E−09	19.75	7.93
C1rb	5.82E−10	1.44E−06	106.37	1.54
Gm13394	4.75E−299	1.20E−294	112.27	N/A
Gm18190	2.57E−06	0.002402677	177.90	−1.28

*FDR* false discovery rate, *FC* fold change.

To further explore the rate of validation, we interrogated by qPCR genes with more modest FC (between 1.5 and 6) and, out of the 13 validation tests, 46% confirmed the RNAseq results (Supplementary Table 7).

By comparing gene expression of wild-type and R6/2 mice, we realized that most of the effects observed on gene expression in the diseased animals treated with AAV-WT- and AAV-LAV-BPIFB4 restored, at least partially, gene expression that was altered in the disease model (Fig. 6b). A possible explanation is that most of the modulated genes are dysregulated by HD pathology and that BPIFB4 isoforms are able to revert this process. Furthermore, investigation in cortex and hippocampus showed that modulation of gene expression by WT- and LAV-BPIFB4 is not confined to the striatum (Fig. 6b)

## Discussion

Exceptional longevity is an inherited trait associated with lower incidence of cardiovascular events^30,31^ and with preservation of some brain function^32^. Indeed, ageing is the primary risk factor for most neurodegenerative diseases, including Alzheimer disease (AD) and Parkinson disease (PD)^33^.

Among the neurodegenerative disorders, HD is a rare inherited condition characterized by progressive striatal and cortical degeneration^4,5^. Both in aged brain and in HD, a sustained inflammation often compromises neuronal viability and correlates with the degree of neurodegeneration. The biological role of immune responses might disclose a rapidly emerging field of study for therapeutic intervention. Here, the ability of the BPIFB4 to activate important processes for cellular homeostasis and to finely tune the immune responses^16,17^ prompted us to investigate its role in a neurodegenerative setting.

Here, we reported for the first time the different expression levels of BPIFB4 in normal Q^7/7^ versus mutant Q^111/111^ striatal cells (Fig. 1c, d). Furthermore, we presented previously unrecognized effects of the LAV-variant in restoring the defective proliferation (Fig. 2b) and the survival response of cells exposed to stress (Fig. 2c), probably in an auto-paracrine manner, as the sustained BPIFB4 secretory profile (Fig. 2d) seems to be instrumental to preserve STHdh Q^111/111^ cellular proteostasis upon MG-132 insult. This is in line with our previous reports showing a stress response process facilitated by the LAV-variant consisting in BPIFB4 phosphorylation/activation by stress kinase protein kinase R-like endoplasmic reticulum kinase-PERK, its complexing with 14-3-3 and HSP 90 and calcium/endothelial nitric oxide synthase (eNOS) activation^20,21^.

Furthermore, by increasing the level of the released BPIFB4 protein, LAV- but not WT-variant exerts a protective effect not only in striatal cells but also on co-cultured microglia. Indeed, we showed that through SDF-1 secretion, the LAV-BPIFB4 overexpressing striatal cells in vitro are able to educate microglia to acquire a (CD163 + IL-10^high^) anti-inflammatory phenotype. This is crucial to properly blunt the unbalanced immune responses. Indeed when an insult to the brain occurs, surveilling microglia become activated and initiate a protective innate immunity in the CNS. With continued activation of microglia, prolonged production of inflammatory mediators by microglia may result in chronic inflammation and implicated in further tissue damage^34^. Accordingly, increased microglial activation was detected in the striatum of presymptomatic R6/2 mice^35^. More interestingly, human PET studies revealed a high degree of correlation between the status of microglia activation in the anterior cingulate and prefrontal cortices and both the disease severity and striatal GABAergic neurons loss in HD patients^36^. At the same way, microglia activation was also identified in PET studies undertaken in presymptomatic HD gene carriers. Taken all these studies together, microglial activation is emerging as an early change in HD^37,38^. Through its peculiar cytokine secretory profile^39,40^ microglia may sustain the etiopathology prior to symptoms onset. In this context the ability of LAV-BPIFB4 to finely shape the M1–M2 balance of human microglia in part explains its therapeutic potential.

At both phenotypic and functional levels, a similar effect of LAV-BPIFB4 on mono-macrophage compartment was previously observed in an atherosclerosis mouse model in vivo^23^. Here, the immunoregulatory action of LAV-BPIFB4 gene transfer skewed macrophages toward an M2-resolving phenotype halting the key clinical signs of atherogenic process (lipid deposition, vascular reactivity, plaque instability, chronic inflammation, etc.). For the achievement of these results, BPIFB4 needs to be probably uptaken and to reach high circulating levels. This may reflect the physiological setting in vivo where BPIFB4 levels are increased in serum of LLIs, and its high level classify their health status^18^. Likewise, homozygous LAV carriers have higher peripheral BPIFB4 and increased phosphorylated eNOS in circulating mononuclear cells^20,21^. Noteworthy, significantly lower plasma BPIFB4 was detected in patients with pathological carotid stenosis (>25%) and intima media thickness >2 mm. Taking all these in account, in future it would be interesting to associate elevated plasma BPIFB4 levels with the HD clinical features to serve as putative prognostic biomarker of the disease state.

From a mechanistic point of view, using CXCR4 inhibitor, we demonstrated that SDF-1/CXCR4 axis plays a critical role in LAV-BPIFB4 beneficial effects both in vitro (Fig. 3) and in vivo (Fig. 5). Even though the mechanism by which LAV-BPIFB4 induces SDF-1 expression is still unknown, the relevance covered by the SDF-1 in myeloid cell differentiation, tissue repair and stem cell mobilization, might in part explain the beneficial effect achieved by LAV-BPIFB4 gene therapy in the context of HD. In particular, SDF-1 enhances the expression of pathogen recognition receptors CD14 and CD163 and induces the secretion of angiogenic and immunosuppressive factors in human peripheral monocytes^29^; accumulating evidence demonstrates that SDF-1 has also major role in the recruitment and retention of CXCR4 + bone marrow-derived cells to the neoangiogenic niches supporting revascularization of ischemic tissue^41^. Finally recent findings support that SDF-1 functions as an anti-inflammatory chemokine that may suppress the antigen-specific immune responses^42^.

The necessity of additional studies in the near future is indeed strengthened by the incoming evidence of a protective role of SDF-1 in neurodegeneration. Stromal cell-derived factor-1α decreases β-amyloid deposition in Alzheimer’s disease mouse model by enhancing microglial association with Aβ deposits^43^. Furthermore, considering the vital roles of neuronal stem cells (NSCs) during tissue repair, SDF-1 is predicted to greatly contribute to the recruitment of NSCs to damaged regions to enhance recovery in PD, AD, and HD. Consistent with this activity, in a viral model of MS, SDF-1-CXCR4 signaling has been seen to mediate NSCs-based remyelination through the regulation of NSC activation and recruitment of neuronal progenitor cells^44^. Accordingly, our findings clearly demonstrated that LAV-BPIFB4 gene therapy is therapeutically effective in R6/2 mice^45^, which recapitulates several futures of human pathology^46–49^. A single injection of AAV-LAV-BPIFB4 protected manifest R6/2 mice from the characteristic progressive motor deficit and prevented the classical body weight loss in the same mice. Biochemical and neuropathological analyses revealed that amelioration of motor behavior in treated R6/2 mice well correlated with increased levels of BDNF in the cortex and with a dramatic reduction of EM48-positive mHtt aggregates in the striatum (Fig. 4). This, along with the better proliferative and survival response to the proteasome inhibition in LAV-BPIFB4-treated STH*dh* Q^111/111^ cells (Fig. 2b, c), confirms the potential of LAV-BPIFB4 to break down mutant protein toxicity. To try to explain the LAV-BPIFB4-mediated pro-survival ability, it has been demonstrated that, through a cAMP-dependent pathway that acts through protein kinase A and MAP kinase, SDF-1 provides generalized trophic support to neurons during their development and maturation^50^. Interestingly, in our KEGG pathway analysis the only category that emerged was HD in the comparison between AAV-LAV-BPIFB4- and AAV-GFP-treated mice, while between AAV-LAV- and AAV-WT-BPIFB4 the highest score has been reached by cAMP signaling pathway (Fig. 6). Collectively, our evidence corroborates the specificity of the favorable connection between LAV-, but not WT-BPIFB4, and SFD-1/CXCR4 signaling pathway in HD. In near future, given the dual action both on striatum and immune surveillance, we propose that the gene transfer or alternatively the usage of a human recombinant protein of LAV-BPIFB4 in presymptomatic HD carriers may represent a promising strategy to improve HD symptoms or delay its onset.

## Materials and methods

### Lentivirus production and titration

BPIFB4 cDNA (WT and LAV isoforms) was cloned from pRK5 expression plasmid^15^ into the lentiviral vector pCDH-EF1-MSC-pA-PGK-copGFP-T2A-Puro (System Biosciences). Lentivirus was produced by transfection of pCDH construct along with the packaging vectors pMD2.VSV.G, pRSV-REV, and pMDLg/pRRE (kindly provided by Prof. Luigi Naldini group, San Raffaele Scientific Institute, Milan, Italy) into HEK293T cells by calcium phosphate transfection. Lentivirus was concentrated by ultracentrifugation (25,000 rpm, 4 h, 4 °C) and stored at −80 °C until immediately prior to use. Virus titration was performed by transducing HEK293T cells with concentrated virus in presence of 4 μg/ml polybrene and measuring GFP expression after 3 days by flow cytometry.

### Cell lines, culture condition, lentiviral infection, and conditioned media

Immortalized striatal cells (ST*Hdh*) derived from a knock-in transgenic mouse containing homozygous Htt loci with a humanized Exon1 with either short (STHdh Q^7/7^) or long polyQ repeats (STHdh Q^111/111^) were grown at 33 °C in 10% FBS in DMEM. Properly authenticated cells were purchased from the Coriell Cell Repositories (Coriell Institute for Medical Research, Camden, NJ, USA).

STHdh Q^111/111^ cells were infected with empty lentiviral vector or particles encoding either WT- or LAV-BPIFB4 (at one multiplicity of infection, two consecutive infections after 24 h in the presence of 4 μg/ml polybrene). After 72 h, cells were selected with 2 µg/ml puromycin for 48 h. BPIFB4 expression was tested by real-time PCR and WB.

In order to obtain the CM, DMEM-FCS medium was discarded, the cells were washed three times and subsequently cultured for 72 h. After that the media from both STHdh Q^7/7^ and STHdh Q^111/111^ were collected and stored in aliquots frozen. This medium was considered CM and used at 20% for subsequent treatments.

Properly authenticated cells HM-SV40 (Cat. No.: T0251) cell lines were purchased from Applied Biological Materials Inc (Richmond, QC, Canada). HM-SV40 were grown in Prigrow III media containing 10% FCS and antibiotics in Applied Biological Materials Inc (Richmond, QC, Canada) in Corning T75 coated flask with collagen I at 0.1 mg/mL (Cat. No.: A10483-01—Gibco Life Technologies Corporation, Grand Island, NY, USA) concentration. Briefly, flasks were treated with collagen 0.1 mg/mL in PBS with calcium and magnesium (Cat. No.: D1283—Sigma-Aldrich, St.Louis, MO, USA) for 1 h at 37 °C; after that, two washes in PBS with calcium and magnesium were needed and flasks were used to seed the cells. Cells were splitted at the 80% of the confluence.

### Cell viability assays

Cell viability was determined using commercially available kit (Cell Counting kit-8, Dojindo) following the provider’s instructions. In the last 1–4 h of treatment 20 μL of CCK-8 solution was added to 200 μL of cell suspension seeded in a 96-well plate and incubated at 37 °C. Absorbance at 450 nm was measured using a microplate reader.

### Determination of STHdh Q^7/7^ and STHdh Q^111/111^ cell proliferation

STHdh Q^7/7^or STHdh Q^111/111^ cells (6 × 103/well) were cultured for 24 h into 96-well plates before addition of MG-132 at the indicated concentration and cultured for additional 24 h at 37 °C. Cell proliferation was evaluated by measuring BrdU incorporation into DNA (BrdU colorimetric assay kit; Roche Applied Science, South San Francisco, CA, USA). Newly synthesized BrdU-DNA was determined by an ELISA plate reader (ThermoScientific) at 450 nm. All experiments were performed in triplicate, and the relative cell growth was expressed as a percentage in comparison with the untreated control cells (100%).

### Cell lysate and western blotting

For preparation of lysates, STHdh cells were trypsinized and collected by centrifugation at 200 × *g* for 5 min. The pellet was washed once with cold DPBS (Gibco^®^, Thermo Fisher Scientific), centrifuged again and lysed in RIPA buffer (50 mM Tris pH 7.5, 150 mM NaCl, 0.5% Triton X-100, 0.5% deoxycholic acid, 10 mg/ml leupeptin, 2 mM phenylmethanesulfonyl fluoride, 10 mg/ml aprotinin, containing protease and phosphatase inhibitors) for 45 min on ice. Afterwards, samples were centrifuged at 14,000 × *g* for 20 min at 4 °C. Supernatant was collected and protein concentrations of RIPA lysates were determined spectrophotometrically using Bradford reagent (Bio-Rad Laboratories). Western blot analysis was performed following standard procedures^51^. 30 μg of protein was separated electrophoretically using 10% SDS-polyacrylamide gel (Bio-Rad). Proteins were transferred on 0.2 μm Trans-Blot^®^ Turbo™ Mini Nitrocellulose membrane (Bio-Rad) using a Trans-Blot^®^ Turbo™ Transfer System (Bio-Rad #1704150). Membrane was blocked with 5% milk (Bio-Rad, Richmond, CA, USA) in TBS containing 0.1% Tween-20 (TBST) at room temperature for 1 h and probed overnight at 4 °C with the following primary antibodies: mouse anti-β-actin (1:30,000; Ab49900, Abcam), rabbit anti-BPIFB4 (1:1000, custom made), mouse anti-p-BPIFB4 (1:500, custom made), rabbit anti-BDNF (Santa Cruz, sc-546). For cortex, reversible Ponceau staining was applied to check equal loading of material under different conditions, as documented elsewhere^52^. Afterwards, membrane was incubated at room temperature for 1 h with the respective secondary antibodies. The immunoblots were visualized by using the enhanced chemiluminescence method using Amersham ECL.

### Enzyme-linked immunosorbent assay

STHdh-conditioned media BPIFB4 levels were determined using Human Long palate, lung, and nasal epithelium carcinoma-associated protein 4 (C20orf186) ELISA kit (Cusabio CSBYP003694HU) following the manufacturer’s protocol. Briefly, conditioned media were incubated for 2 h at 37 °C in the assay coated microplate. After removing any unbound substances, a biotin-conjugated antibody specific for C20orf186 was added to the wells and incubated for 1 h at 37 °C. After washing, avidin-conjugated horseradish peroxidase (HRP) was added to the wells and incubated for 1 h at 37 °C. Following a wash, substrate solution was added and the consequent color development was stopped. Optical density was measured at 450 nm.

STHdh-conditioned media SDF-1 levels were determined using DuoSet^®^ ELISA Development Systems (R&D systems) following the manufacturer’s protocol. First, a 96-well microplate was coated with diluted capture antibody overnight at room temperature. After washing, plate was blocked by reagent diluent and incubated for 1 h at RT. Following washes, conditioned media were first incubated for 2 h at RT and then detection antibody was added and incubated for 2 h a RT. After washing, streptavidin-HRP was added for 20 min at RT. Substrate solution was added and the optical density of each well was determined using a microplate reader set to 450 nm.

STHdh-conditioned media IL-10 levels were determined using a beads-based multiplex ELISA (LEGENDplex, Biolegend, USA). Conditioned media were incubated for 2 h with the beads and detection antibodies, followed by 30 min incubation with SA-PE. After washing, beads were resuspended in washing buffer and acquired using a FACS VERSE flow cytometer (BD Biosciences). Data were analyzed with the LEGENDplex Data Analysis Software.

### Antibodies and flow cytometry

Cell suspensions were stained with mAb against human CD163 (REA812; Miltenyi Biotec GmbH; 1:50). After 20 min incubation at 4 °C in the dark, cells were washed, centrifugated, and resuspended in staining buffer for the FACS analysis. For each test, cells were analyzed using FACS Verse Flow Cytometer (BD Biosciences).

### Animal models

Breeding pairs of the R6/2 line of transgenic female mice [strain name: B6CBA-tgN (HDexon1) 62Gpb/1J] with ∼160 ± 10 (CAG) repeat expansions were purchased from the Jackson Laboratories. Male R6/2 mice were crossed with female B6CBA WT mice for colony maintenance. All procedures on animals were approved by the IRCCS Neuromed Animal Care Review Board and were conducted according to EU Directive 2010/63/EU for animal experiments. In vivo experiments were carried out in both R6/2 female mice and WT littermates at 7 weeks of age. To ensure homogeneity of experimental cohorts, mice from the same F generation were assigned to experimental groups, such that age and weight were matched. An overall well-being of each mouse was, however, the selective inclusion criteria. No specific exclusion criteria were applied. Only if accidental events (such jumping from the Rotarod apparatus) occurred during the behavioral tests, animals were excluded from the analysis. All criteria were preestablished.

Power analysis has been conducted to determine adequate number of mice to measure motor signs and animal behavioral in a statistically significant manner as previously described^53,54^. Software G Power for sample size calculation has been also used^55^. In order to reduce the number of animals used for this study, where possible, multiple biochemical analyses, evaluation and record of motor function were conducted on the same group of mice. The investigators were blinded to group allocation during data collection and analysis

### Infection of mice with AAV

WT and R6/2 mice weighing ~25 g were used in this study. All animals were randomly divided into the control and treated groups and quantification of the results was performed by second individual who was blind to the genotype of the animal and/or the hypothesis that was being tested for each group.

The mice were placed individually in an induction chamber and anaesthesia induced with 5% isoflurane in 100% O_2_ (delivery rate, 5 L/min) until loss of righting reflex. After induction, the mice were placed in dorsal recumbence on a homeothermic blanket (N-HB101-S-402) to maintain body temperature at 37 °C. Anaesthesia was maintained with 1% isoflurane in 100% O_2_ at 1.5 L/min, administered by means of a facemask connected to a coaxial circuit (Fluovac anaesthetic mask). The procedures replicated typical clinical practice when laboratory mice are anaesthetized for surgical procedures. Vascular surgery was performed with the aid of a microscope at ×2–10 magnification. Femoral arteries were exposed and isolated circumferentially from the inguinal ligament to the knee; all side branches were ligated. To obtain an isolated arterial segment, the superficial femoral artery was first controlled proximally with two microvascular clips. After temporary clamping of the proximal and distal femoral arteries, either 100 µl of saline alone or saline plus AAV-GFP, AAV-WT-BPIFB4, or AAV-LAV-BPIFB4 was infused into the femoral artery and incubated for 15 min. Viral titre was 1 × 10^13^ GC/kg for each experimental condition. After incubation, the distal femoral artery was permanently ligated, and clamps on the proximal femoral artery were removed to restore femoral blood flow. Mice remained anaesthetized for 1 h, after which all received 100% O_2_ until recovery of righting reflex. Animal groups treated with CXCR4 inhibitor received a daily intraperitoneally injection of AMD3100 at the concentration of 5 mg/kg until the end of treatment.

### Analysis of motor behavior

Fine-motor skills and coordination were performed using well-validated motor tests according to the standard recommendations. All tests took place during the light phase of the light–dark cycle. Six to nine mice per experimental group were used in each test. All mice received training for 2 consecutive days on each instrument and task before performing motor behavior measurements. Before training and testing, mice underwent a period of habituation to the testing room and equipment. Motor coordination and balance were tested on a Rotarod apparatus (Ugo Basile) as previously described^56^. Briefly, mice were tested at fixed speed (20 rpm) on the Rotarod for 1 min. Each mouse was tested in three consecutive trials of 1 min each, with 1 min rest between trials. The time spent on the Rotarod in each of the three trials was averaged to give the overall time for each mouse.

Skilled walking, limb placement, and limb coordination were all assessed by the ladder rung walking task as previously described^56^. All tests were carried out once a week until the 11th week of age. Concomitant with the analysis of motor performance, animal body weight was also measured. All mice were examined daily to determine disease progression and overall well-being.

### Immunohistochemistry for mutant HTT aggregation

Wild-type and R6/2 mice were sacrificed by cervical dislocation. Brains were removed and trimmed by removing the olfactory bulbs and spinal cord. The remaining brain was weighed, processed, and embedded in paraffin wax, and 10 mm coronal sections cut on an RM 2245 microtome (Leica Microsystems). Three or four mice/group were used, and four coronal sections spread over the anterior–posterior extent of the brain (200–300 µm intersection distance) were scanned. Immunostaining for mutant HTT aggregates was carried out by using EM48 antibody (1:100) (Millipore) as previously described^57^. The average area of striatal mHTT aggregates per section for each mouse brain was quantified with ImageJ software (developed by Wayne Rasband, National Institutes of Health, USA) and reported as aggregate area (μm^2^)^56^.

### RNA extraction and expression analysis by qPCR

RNA was extracted from striatum, cortex and hippocampus tissue, and STHdh Q^111/111^ cells using the small RNA miRNeasy Mini Kit (Qiagen) and quantified by Nanodrop Spectrophotometer. Genomic DNA was removed by DNase I treatment. Reverse transcription was performed on 1 μg of total RNA using SuperScript VILO cDNA Synthesis Kit (Thermo Fisher Scientific) real-time PCR. Real-time PCR was performed by using primers listed in Table 2 with Sybr Green PCR Master Mix (Applied Biosystem), and the results were determined with QuantStudio 6 Flex detection system (Applied Biosystems). Reaction mixtures (10 μl) included 10 ng of cDNA and 250 nM concentrations of primers in the reaction buffer and enzyme supplied by the manufacturer. All reactions were performed in triplicate, including negative control samples, which never showed significant threshold cycles (CT). The relative amounts of the transcripts were determined with 18S rRNA as the reference gene ([CT (gene of interest) − CT(16 S)] = ΔCT).

**Table 2:** Real-time PCR primers used in gene expression validation experiment.

Gene	Primers	Position
Hba-a2	Fw: GGATCCCGTCAACTTCAAGC Rev: CAAGGGAGAGAAGAAGGGCA	chr11:32284110–32284442
Pttg1	Fw: TGCTCCTGATGATGCCTACC Rev: ATGAGAGGCACGCCATTCA	chr11:43421196–43422948
9430037G07Rik	Fw: GCAGAAGTGCATGACCAGTGAC Rev: CATACAGGAATCAACTCTGCAGG	chr9:88598060–88598480
Bfsp2	Fw: GTCCTTGAGACGATCCGAGTTC Rev: GATACGTGGACCACCTCTGTCT	chr9:103432765–103448629
Gm8098	Fw: CAGCAGCAGCGTCATGC Rev: GATACGTGGACCACCTCTGTCT	chr11:30267849–30279571
Eps8l1	Fw: CATGTCAATCACCTGGTCACC Rev: AGTTCTTCCTTGGAGACTGGATC	chr7:4469246–4470290
Gm26793	Fw: GGAGCTCTGGGAGAATGTCA Rev: TCCCTGGTACCCCACATTTA	chr7:144895195–144895424
C1rb	Fw: ATCAGGCGCTACTGTCCTTCAC Rev: TCCCTGGTACCCCACATTTA	chr6:124577011–124577434
Gm18190	Fw: CCCCACGAACACTGAGAGTA Rev: TTGCCACATTCGCTACACTG	chr7:9789206–9789358
Fgf15	Fw: GTCGCTCTGAAGACGATTGC Rev: TCCATGCTGTCACTCTCCAG	chr7:144897136–144899887

*FDR* false discovery rate, *FC* fold change.

### RNA sequencing analysis

For the preparation of indexed libraries (TruSeq Stranded mRNA) and paired-end sequencing in single (2 × 150, ~30,000,000 total reads/sample) on Illumina platform Hiseq 2500, as first samples were assessed after dilution by using Nanodrop spectrophotometer (to evaluate purity) and TapeStation 4200 (to evaluate integrity) as better detailed in supplementary quality control analysis report (Supplementary Material online).

Fastq underwent to Quality Control using FastQC tool (www.bioinformatics.babraham.ac.uk). To remove the adapter sequences, cutadapt was used^58^. The mapping of paired-end reads was performed using STAR (Version 2.5.2b)^59^ on reference GRCm38/mm10 assembly obtained from Ensembl^60^. The quantification of transcripts expressed for each sample was performed using FeatureCount^61^ algorithm. EdgeR^62^ was used to perform the normalization matrix of all samples and the differentially expression analysis. To perform Gene Ontology analysis Panther database was used (Version 13.1)^63^.

### Statistical analysis

In all experiments shown, statistical analysis was performed by using the GraphPad prism 6.0 software for Windows (GraphPad software). For each type of assay or phenotypic analysis, data obtained from multiple experiments are calculated as mean ± SD and analyzed for statistical significance using appropriate tests. In analysis of variance (ANOVA) for multiple comparison *P* values < 0.05 were considered significant; *P < 0.05, **P < 0.01, and ***P < 0.001. Two-way ANOVA followed by Bonferroni posttest was used to compare treatment groups with the horizontal ladder task and rotarod tests as well as for the analysis of body weight.

## Supplementary information

Supplementary information

Supplementary information 2

Supplementary information 3

Supplementary information 4

Supplementary information 5

Supplementary information 6

Supplementary information 7

Supplementary information 8

Supplementary information 9
