# Supplementary figures and images for: The longevity-associated variant of BPIFB4 improves a CXCR4-mediated striatum–microglia crosstalk preventing disease progression in a mouse model of Huntington’s disease
Source: Cell Death Dis. 2020 Jul 18;11(7):546. doi: 10.1038/s41419-020-02754-w (PMC7368858; doi:10.1038/s41419-020-02754-w)

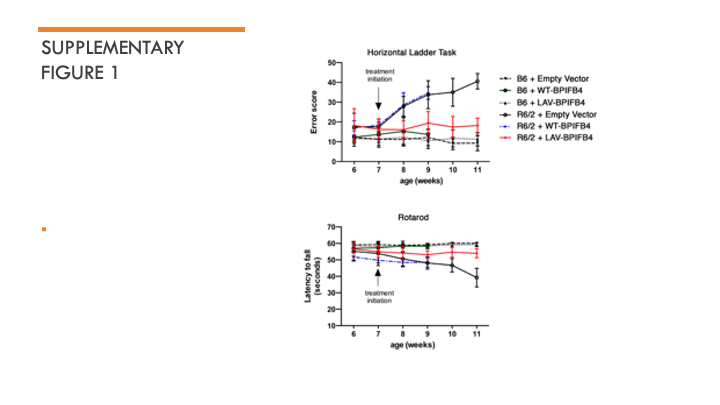

Supplement: Supplementary file 1 — Supplementary information [file 41419_2020_2754_MOESM1_ESM.tif]

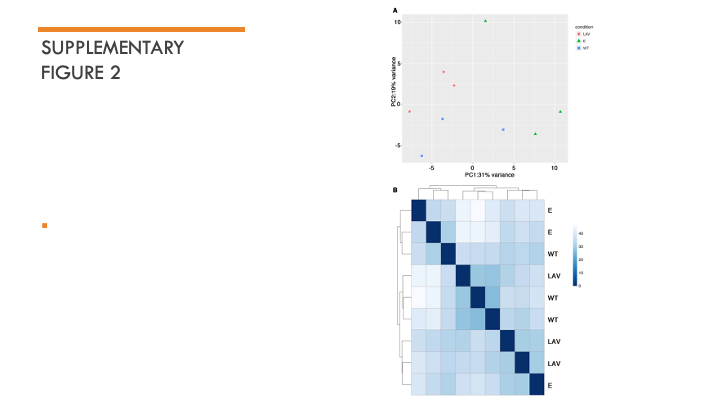

Supplement: Supplementary file 2 — Supplementary information 2 [file 41419_2020_2754_MOESM2_ESM.tif]
