# Supplementary material for: The longevity-associated variant of BPIFB4 improves a CXCR4-mediated striatum–microglia crosstalk preventing disease progression in a mouse model of Huntington’s disease
Source: Cell Death Dis. 2020 Jul 18;11(7):546. doi: 10.1038/s41419-020-02754-w (PMC7368858; doi:10.1038/s41419-020-02754-w)
Supplement: Supplementary file 3 — Supplementary information 3 [file 41419_2020_2754_MOESM3_ESM.docx]

| **Supplementary table 1**  **Differential expression (RNAseq analysis) Empty Vector Vs LAV-BPIFB4 (FDR<=5)** | | | | | |
| --- | --- | --- | --- | --- | --- |
|  |  |  |  |  |  |
| **Gene** | **logFC** | **FoldChange** | **Fold-Change** | **PValue** | **FDR** |
| Hba-a2 | -4,86 | 0,03 | -29,04 | 6,97E-20 | 4,40E-16 |
| Pttg1 | -4,68 | 0,04 | -25,58 | 2,24E-74 | 2,82E-70 |
| Gm37460 | -4,58 | 0,04 | -23,92 | 1,73E-07 | 0,000256779 |
| 9430037G07Rik | -4,41 | 0,05 | -21,22 | 0,00012483 | 0,049276587 |
| Bfsp2 | -3,79 | 0,07 | -13,80 | 6,26E-10 | 1,44E-06 |
| Gm8098 | -2,94 | 0,13 | -7,67 | 3,42E-12 | 1,44E-08 |
| Dnah3 | -2,52 | 0,17 | -5,74 | 8,72E-05 | 0,037999653 |
| Dnah12 | -2,51 | 0,18 | -5,71 | 1,70E-06 | 0,001945821 |
| E230008N13Rik | -2,28 | 0,21 | -4,85 | 4,15E-05 | 0,023160311 |
| Wdr63 | -2,25 | 0,21 | -4,76 | 6,54E-06 | 0,005008415 |
| Hydin | -2,19 | 0,22 | -4,57 | 4,65E-06 | 0,003787325 |
| Gm10654 | -2,18 | 0,22 | -4,52 | 6,74E-05 | 0,032761147 |
| Dnah10 | -2,17 | 0,22 | -4,51 | 2,50E-05 | 0,016202268 |
| Daw1 | -2,13 | 0,23 | -4,37 | 3,74E-05 | 0,021808528 |
| Thbs4 | -1,91 | 0,27 | -3,75 | 1,06E-09 | 2,24E-06 |
| Ccdc81 | -1,89 | 0,27 | -3,71 | 2,65E-05 | 0,016759925 |
| Mki67 | -1,88 | 0,27 | -3,69 | 3,07E-06 | 0,002671976 |
| Slc30a2 | -1,81 | 0,28 | -3,51 | 8,39E-05 | 0,037201406 |
| Top2a | -1,69 | 0,31 | -3,23 | 5,40E-06 | 0,004266439 |
| Lrrc48 | -1,53 | 0,35 | -2,89 | 1,77E-06 | 0,001945821 |
| Cenpf | -1,52 | 0,35 | -2,87 | 2,93E-05 | 0,01806571 |
| Vwa3a | -1,42 | 0,37 | -2,67 | 8,57E-07 | 0,00120312 |
| Ppl | -1,39 | 0,38 | -2,62 | 2,86E-08 | 5,15E-05 |
| Uhrf1 | -1,24 | 0,42 | -2,36 | 0,000103325 | 0,042793357 |
| Irgm2 | -1,09 | 0,47 | -2,12 | 6,82E-06 | 0,005065738 |
| Dlx2 | -0,93 | 0,53 | -1,90 | 1,26E-05 | 0,008820524 |
| Mlh1 | -0,92 | 0,53 | -1,89 | 3,63E-05 | 0,021808528 |
| Hmgb2 | -0,87 | 0,55 | -1,83 | 2,50E-05 | 0,016202268 |
| Slc5a5 | -0,85 | 0,56 | -1,80 | 7,97E-05 | 0,036516944 |
| Igfbp5 | -0,84 | 0,56 | -1,79 | 9,84E-05 | 0,041551904 |
| Mxra8 | -0,83 | 0,56 | -1,77 | 7,14E-05 | 0,033568345 |
| Crym | -0,80 | 0,58 | -1,74 | 2,82E-06 | 0,002546113 |
| Col11a1 | -0,75 | 0,59 | -1,68 | 8,09E-05 | 0,036516944 |
| C4b | -0,69 | 0,62 | -1,61 | 6,76E-09 | 1,31E-05 |
| Ublcp1 | -0,57 | 0,68 | -1,48 | 3,86E-06 | 0,003251832 |
| Scoc | -0,50 | 0,71 | -1,42 | 1,81E-05 | 0,012349492 |
| Dlgap3 | 0,41 | 1,33 | 1,33 | 0,000120392 | 0,048279186 |
| mt-Nd1 | 0,47 | 1,38 | 1,38 | 5,86E-05 | 0,029626544 |
| Trnp1 | 0,52 | 1,43 | 1,43 | 2,31E-06 | 0,002239896 |
| Scg5 | 0,54 | 1,45 | 1,45 | 2,08E-06 | 0,002171901 |
| Kif17 | 0,54 | 1,45 | 1,45 | 6,54E-05 | 0,032388047 |
| Ccdc85b | 0,54 | 1,45 | 1,45 | 5,83E-05 | 0,029626544 |
| Ppp1r1b | 0,56 | 1,48 | 1,48 | 4,22E-05 | 0,023160311 |
| C1qtnf4 | 0,58 | 1,49 | 1,49 | 4,46E-05 | 0,02395825 |
| Tesc | 0,61 | 1,53 | 1,53 | 7,29E-06 | 0,005265312 |
| Dhrs7b | 0,67 | 1,59 | 1,59 | 7,17E-05 | 0,033568345 |
| Tmem158 | 0,71 | 1,64 | 1,64 | 1,77E-06 | 0,001945821 |
| Pcsk1n | 0,71 | 1,64 | 1,64 | 9,46E-07 | 0,001257766 |
| Lrrc10b | 0,72 | 1,65 | 1,65 | 1,73E-07 | 0,000256779 |
| Scand1 | 0,99 | 1,98 | 1,98 | 2,15E-06 | 0,002171901 |
| Gm6969 | 1,12 | 2,17 | 2,17 | 5,31E-11 | 1,49E-07 |
| Snora7a | 1,54 | 2,91 | 2,91 | 4,57E-05 | 0,024062769 |
| Gm23935 | 1,55 | 2,92 | 2,92 | 1,17E-07 | 0,00019649 |
| Xlr3a | 1,82 | 3,53 | 3,53 | 3,80E-05 | 0,021808528 |
| 9330104G04Rik | 1,94 | 3,83 | 3,83 | 1,29E-06 | 0,001634459 |
| Gm12663 | 2,13 | 4,39 | 4,39 | 2,23E-11 | 8,03E-08 |
| Pglyrp1 | 2,14 | 4,40 | 4,40 | 2,70E-11 | 8,54E-08 |
| Gm6304 | 2,35 | 5,11 | 5,11 | 0,000115504 | 0,047066214 |
| Gm7335 | 3,08 | 8,47 | 8,47 | 7,05E-23 | 5,94E-19 |
| Eps8l1 | 3,62 | 12,28 | 12,28 | 9,87E-05 | 0,041551904 |
| Gm26793 | 4,30 | 19,75 | 19,75 | 3,08E-13 | 1,56E-09 |
| C1rb | 6,73 | 106,37 | 106,37 | 5,82E-10 | 1,44E-06 |
| Gm13394 | 6,81 | 112,27 | 112,27 | 4,75E-299 | 1,20E-294 |
| Gm18190 | 7,47 | 177,90 | 177,90 | 2,57E-06 | 0,002402677 |
