# Supplementary material for: The longevity-associated variant of BPIFB4 improves a CXCR4-mediated striatum–microglia crosstalk preventing disease progression in a mouse model of Huntington’s disease
Source: Cell Death Dis. 2020 Jul 18;11(7):546. doi: 10.1038/s41419-020-02754-w (PMC7368858; doi:10.1038/s41419-020-02754-w)
Supplement: Supplementary file 4 — Supplementary information 4 [file 41419_2020_2754_MOESM4_ESM.docx]

| **Supplementary Table 2**  **Differential expression (RNAseq analysis) WT-BPIFB4 Vs LAV-BPIFB4 (FDR<=5)** | | | | | |
| --- | --- | --- | --- | --- | --- |
|  |  |  |  |  |  |
| **Gene** | **logFC** | **FoldChange** | **Fold-Change** | **PValue** | **FDR** |
| Nrn1 | -1,34 | 0,39 | -2,53 | 2,80E-05 | 0,027352747 |
| Fos | -1,19 | 0,44 | -2,28 | 6,67E-07 | 0,001821572 |
| Chrna4 | -1,02 | 0,49 | -2,02 | 3,43E-05 | 0,03085014 |
| Nr4a1 | -0,93 | 0,52 | -1,91 | 2,21E-09 | 3,12E-05 |
| Crym | -0,83 | 0,56 | -1,78 | 7,17E-07 | 0,001821572 |
| Serpina3n | -0,78 | 0,58 | -1,71 | 1,10E-06 | 0,002328937 |
| Gfra2 | -0,73 | 0,60 | -1,66 | 5,36E-05 | 0,041290137 |
| Kcnc2 | -0,63 | 0,65 | -1,54 | 8,05E-06 | 0,01137167 |
| 6330403K07Rik | -0,61 | 0,66 | -1,53 | 6,29E-05 | 0,044404887 |
| Syt13 | -0,61 | 0,66 | -1,53 | 2,10E-07 | 0,000887897 |
| Junb | -0,59 | 0,67 | -1,50 | 3,80E-05 | 0,031550398 |
| Cpne5 | 0,43 | 1,35 | 1,35 | 5,99E-05 | 0,043486511 |
| Ppp1r1b | 0,47 | 1,39 | 1,39 | 2,51E-05 | 0,027352747 |
| Drd2 | 0,49 | 1,40 | 1,40 | 4,88E-05 | 0,038792295 |
| Wscd2 | 0,49 | 1,41 | 1,41 | 2,70E-05 | 0,027352747 |
| Kremen1 | 0,50 | 1,41 | 1,41 | 3,85E-05 | 0,031550398 |
| Mme | 0,51 | 1,42 | 1,42 | 5,43E-06 | 0,008633232 |
| Gcnt2 | 0,54 | 1,45 | 1,45 | 3,35E-05 | 0,03085014 |
| Adora2a | 0,54 | 1,46 | 1,46 | 3,52E-05 | 0,03085014 |
| Gpr88 | 0,55 | 1,47 | 1,47 | 1,97E-06 | 0,003584385 |
| Inf2 | 0,55 | 1,47 | 1,47 | 8,51E-06 | 0,011391864 |
| Cnr1 | 0,57 | 1,48 | 1,48 | 1,92E-05 | 0,024354737 |
| Kcng1 | 0,57 | 1,48 | 1,48 | 2,04E-05 | 0,024652267 |
| Rasd2 | 0,58 | 1,50 | 1,50 | 8,32E-07 | 0,001921574 |
| Lpl | 0,60 | 1,51 | 1,51 | 5,61E-07 | 0,001782101 |
| Ano3 | 0,61 | 1,53 | 1,53 | 4,80E-07 | 0,001744821 |
| Actn2 | 0,67 | 1,59 | 1,59 | 6,73E-09 | 4,28E-05 |
| Scn4b | 0,69 | 1,61 | 1,61 | 2,46E-09 | 3,12E-05 |
| Lrrc10b | 0,79 | 1,73 | 1,73 | 2,42E-08 | 0,000123155 |
| Frem2 | 0,81 | 1,75 | 1,75 | 2,73E-05 | 0,027352747 |
| Gm6304 | 3,02 | 8,11 | 8,11 | 2,60E-05 | 0,027352747 |
| Gm26793 | 3,33 | 10,06 | 10,06 | 3,78E-09 | 3,21E-05 |
| Eps8l1 | 3,47 | 11,08 | 11,08 | 6,30E-06 | 0,009426334 |
| Gm4875 | 4,21 | 18,48 | 18,48 | 1,33E-06 | 0,002594136 |
| Gm6612 | 5,97 | 62,89 | 62,89 | 5,76E-05 | 0,043081284 |
| Gm18190 | 7,32 | 159,46 | 159,46 | 4,12E-06 | 0,006973729 |
