# Supplementary material for: The longevity-associated variant of BPIFB4 improves a CXCR4-mediated striatum–microglia crosstalk preventing disease progression in a mouse model of Huntington’s disease
Source: Cell Death Dis. 2020 Jul 18;11(7):546. doi: 10.1038/s41419-020-02754-w (PMC7368858; doi:10.1038/s41419-020-02754-w)
Supplement: Supplementary file 5 — Supplementary information 5 [file 41419_2020_2754_MOESM5_ESM.docx]

| **Supplementary table 3**  **Differential expression (RNAseq analysis) WT-BPIFB4 Vs Empty Vector (FDR<=5)** | | | | | |
| --- | --- | --- | --- | --- | --- |
|  |  |  |  |  |  |
| **Gene** | **logFC** | **FoldChange** | **Fold-Change** | **PValue** | **FDR** |
| Shox2 | -2,41 | 0,19 | -5,30 | 0,000117049 | 0,048725024 |
| Tspan11 | -1,89 | 0,27 | -3,70 | 0,000107345 | 0,04776654 |
| Gm12892 | -1,33 | 0,40 | -2,51 | 7,00E-05 | 0,036252438 |
| Rpsa-ps10 | -1,04 | 0,49 | -2,05 | 3,70E-08 | 0,000155799 |
| Ifi27l2a | -0,99 | 0,50 | -1,99 | 6,82E-05 | 0,036035595 |
| Resp18 | -0,97 | 0,51 | -1,95 | 1,16E-05 | 0,012270371 |
| Nr4a1 | -0,81 | 0,57 | -1,75 | 1,80E-05 | 0,016802634 |
| Sac3d1 | -0,80 | 0,57 | -1,74 | 2,86E-05 | 0,02074099 |
| Hmgn2 | -0,72 | 0,61 | -1,65 | 6,08E-07 | 0,001298499 |
| Coprs | -0,68 | 0,62 | -1,61 | 1,85E-05 | 0,016802634 |
| Srp9 | -0,68 | 0,62 | -1,60 | 5,05E-05 | 0,03123505 |
| Nnat | -0,64 | 0,64 | -1,56 | 7,71E-06 | 0,011498794 |
| Rps27 | -0,63 | 0,64 | -1,55 | 5,84E-05 | 0,03415508 |
| Rps29 | -0,60 | 0,66 | -1,52 | 0,000117183 | 0,048725024 |
| Smyd2 | -0,57 | 0,67 | -1,48 | 1,08E-05 | 0,012270371 |
| Selm | -0,56 | 0,68 | -1,48 | 5,79E-05 | 0,03415508 |
| Podxl2 | -0,55 | 0,68 | -1,47 | 1,16E-05 | 0,012270371 |
| Cox5b | -0,55 | 0,69 | -1,46 | 8,58E-06 | 0,012087465 |
| Uqcrh | -0,54 | 0,69 | -1,45 | 7,37E-05 | 0,036671781 |
| Pdxdc1 | -0,54 | 0,69 | -1,45 | 0,000125306 | 0,049580427 |
| Rpl28 | -0,53 | 0,69 | -1,44 | 4,39E-05 | 0,028369105 |
| Iscu | -0,51 | 0,70 | -1,43 | 3,03E-05 | 0,021372257 |
| Ndufa12 | -0,50 | 0,71 | -1,41 | 6,11E-05 | 0,034258074 |
| Comt | -0,49 | 0,71 | -1,41 | 0,000101961 | 0,046181043 |
| Rell2 | -0,47 | 0,72 | -1,39 | 7,73E-05 | 0,037709318 |
| Strn | 0,51 | 1,42 | 1,42 | 0,000115056 | 0,048725024 |
| Ano3 | 0,52 | 1,43 | 1,43 | 2,47E-05 | 0,019579748 |
| Inf2 | 0,53 | 1,45 | 1,45 | 2,69E-06 | 0,004876617 |
| RP24-68G17.4 | 0,57 | 1,48 | 1,48 | 5,93E-05 | 0,03415508 |
| Fnbp1l | 0,59 | 1,51 | 1,51 | 0,00011173 | 0,048725024 |
| Arrdc3 | 0,64 | 1,55 | 1,55 | 3,21E-05 | 0,021587256 |
| U2surp | 0,65 | 1,57 | 1,57 | 2,24E-06 | 0,004371465 |
| Psme4 | 0,66 | 1,58 | 1,58 | 3,03E-06 | 0,005117855 |
| Cpeb4 | 0,66 | 1,58 | 1,58 | 2,76E-05 | 0,02074099 |
| Qk | 0,66 | 1,58 | 1,58 | 9,56E-05 | 0,044117101 |
| Btaf1 | 0,70 | 1,62 | 1,62 | 5,32E-08 | 0,000155799 |
| Utp14b | 0,73 | 1,66 | 1,66 | 0,000131326 | 0,049715846 |
| Vwf | 0,73 | 1,66 | 1,66 | 4,47E-05 | 0,028369105 |
| Sema3e | 0,74 | 1,67 | 1,67 | 5,53E-08 | 0,000155799 |
| Zfp280c | 0,77 | 1,70 | 1,70 | 1,11E-05 | 0,012270371 |
| Xist | 0,79 | 1,73 | 1,73 | 0,000122206 | 0,049580427 |
| Plin4 | 0,81 | 1,75 | 1,75 | 6,14E-07 | 0,001298499 |
| Ide | 0,87 | 1,83 | 1,83 | 1,32E-09 | 8,39E-06 |
| Sema3a | 0,88 | 1,85 | 1,85 | 9,83E-06 | 0,012270371 |
| Gm9938 | 1,02 | 2,03 | 2,03 | 9,57E-05 | 0,044117101 |
| Wdfy1 | 1,04 | 2,05 | 2,05 | 1,98E-05 | 0,017286348 |
| Sspo | 1,05 | 2,08 | 2,08 | 7,23E-05 | 0,036671781 |
| Satb2 | 1,14 | 2,21 | 2,21 | 3,23E-05 | 0,021587256 |
| Ppl | 1,26 | 2,40 | 2,40 | 6,66E-06 | 0,010552534 |
| Gm17066 | 1,36 | 2,57 | 2,57 | 2,40E-05 | 0,019579748 |
| Gm37607 | 1,50 | 2,83 | 2,83 | 4,50E-08 | 0,000155799 |
| RP23-235E15.6 | 1,51 | 2,85 | 2,85 | 6,34E-05 | 0,034258074 |
| Spag16 | 1,59 | 3,01 | 3,01 | 0,000126415 | 0,049580427 |
| Mki67 | 1,78 | 3,44 | 3,44 | 2,83E-05 | 0,02074099 |
| Thbs4 | 1,89 | 3,72 | 3,72 | 9,48E-12 | 1,20E-07 |
| Dnah10 | 2,28 | 4,85 | 4,85 | 0,000129014 | 0,049580427 |
| Hydin | 2,41 | 5,33 | 5,33 | 1,38E-05 | 0,01401924 |
| Gm37310 | 2,47 | 5,52 | 5,52 | 2,09E-05 | 0,017703162 |
| Slc30a2 | 2,48 | 5,59 | 5,59 | 2,11E-07 | 0,000535145 |
| Impg1 | 2,64 | 6,24 | 6,24 | 1,74E-05 | 0,016802634 |
| Bfsp2 | 2,64 | 6,25 | 6,25 | 0,000128685 | 0,049580427 |
| Gm8098 | 2,73 | 6,62 | 6,62 | 9,67E-11 | 8,17E-07 |
| Dnah12 | 2,76 | 6,78 | 6,78 | 6,35E-05 | 0,034258074 |
| Dnah3 | 2,88 | 7,37 | 7,37 | 9,66E-06 | 0,012270371 |
| Pttg1 | 3,97 | 15,62 | 15,62 | 2,62E-08 | 0,000133106 |
| Hba-a2 | 4,50 | 22,65 | 22,65 | 1,48E-27 | 3,76E-23 |
| Gm38255 | 5,52 | 45,75 | 45,75 | 8,80E-05 | 0,042108859 |
