# Supplementary material for: The longevity-associated variant of BPIFB4 improves a CXCR4-mediated striatum–microglia crosstalk preventing disease progression in a mouse model of Huntington’s disease
Source: Cell Death Dis. 2020 Jul 18;11(7):546. doi: 10.1038/s41419-020-02754-w (PMC7368858; doi:10.1038/s41419-020-02754-w)
Supplement: Supplementary file 6 — Supplementary information 6 [file 41419_2020_2754_MOESM6_ESM.docx]

| **GENE ONTOLOGY EMPTY VECTOR VS LAV-BPIFB4** | | | | | | | | | | | | | | | | | | | | | |
| --- | --- | --- | --- | --- | --- | --- | --- | --- | --- | --- | --- | --- | --- | --- | --- | --- | --- | --- | --- | --- | --- |
|  |  |  | |  | |  |  |  | |  |  | |  | |  |  | |  |  | |  |
|  |  |  | |  | |  |  |  | |  |  | |  | |  |  | |  |  | |  |
| **Biological Process Analysis** | | | | | | | | | | | | | | | | | | | | | |
| **Category** | **Term** | | **Count** | | **%** | **PValue** | **LOG(Pvalue)** | | **Genes** | **List Total** | **Pop Hits** | **Pop Total** | | **Fold Enrichment** | | **Bonferroni** | | **Benjamini** | **FDR** |  |  |
| *GOTERM_BP_DIRECT* | *GO:0045143~homologous chromosome segregation* | | *2,00* | | *0,03* | *0,01* | *1,97* | | *MLH1, PTTG1* | *40,00* | *5,00* | *18082,00* | | *180,82* | | *0,96* | | *0,96* | *13,42* |  |  |
| *GOTERM_BP_DIRECT* | *GO:0007018~microtubule-based movement* | | *3,00* | | *0,04* | *0,01* | *1,91* | | *DNAH12, KIF17, DNAH3* | *40,00* | *78,00* | *18082,00* | | *17,39* | | *0,97* | | *0,84* | *15,20* |  |  |
| *GOTERM_BP_DIRECT* | *GO:0007059~chromosome segregation* | | *3,00* | | *0,04* | *0,02* | *1,80* | | *CENPF, PTTG1, TOP2A* | *40,00* | *89,00* | *18082,00* | | *15,24* | | *0,99* | | *0,79* | *19,11* |  |  |
| *GOTERM_BP_DIRECT* | *GO:0006265~DNA topological change* | | *2,00* | | *0,03* | *0,02* | *1,77* | | *HMGB2, TOP2A* | *40,00* | *8,00* | *18082,00* | | *113,01* | | *0,99* | | *0,72* | *20,59* |  |  |
| *GOTERM_BP_DIRECT* | *GO:0045654~positive regulation of megakaryocyte differentiation* | | *2,00* | | *0,03* | *0,02* | *1,77* | | *TESC, HMGB2* | *40,00* | *8,00* | *18082,00* | | *113,01* | | *0,99* | | *0,72* | *20,59* |  |  |
| *GOTERM_BP_DIRECT* | *GO:0016486~peptide hormone processing* | | *2,00* | | *0,03* | *0,02* | *1,63* | | *SCG5, PCSK1N* | *40,00* | *11,00* | *18082,00* | | *82,19* | | *1,00* | | *0,76* | *27,17* |  |  |
| *GOTERM_BP_DIRECT* | *GO:0000712~resolution of meiotic recombination intermediates* | | *2,00* | | *0,03* | *0,03* | *1,53* | | *MLH1, TOP2A* | *40,00* | *14,00* | *18082,00* | | *64,58* | | *1,00* | | *0,78* | *33,20* |  |  |
| *GOTERM_BP_DIRECT* | *GO:0006956~complement activation* | | *2,00* | | *0,03* | *0,03* | *1,50* | | *C1RB, C4B* | *40,00* | *15,00* | *18082,00* | | *60,27* | | *1,00* | | *0,75* | *35,10* |  |  |
| *GOTERM_BP_DIRECT* | *GO:0006259~DNA metabolic process* | | *2,00* | | *0,03* | *0,04* | *1,37* | | *MKI67, TOP2A* | *40,00* | *20,00* | *18082,00* | | *45,21* | | *1,00* | | *0,80* | *43,81* |  |  |
| *GOTERM_BP_DIRECT* | *GO:0021591~ventricular system development* | | *2,00* | | *0,03* | *0,04* | *1,37* | | *HYDIN, CENPF* | *40,00* | *20,00* | *18082,00* | | *45,21* | | *1,00* | | *0,80* | *43,81* |  |  |
| *GOTERM_BP_DIRECT* | *GO:0007049~cell cycle* | | *5,00* | | *0,07* | *0,04* | *1,37* | | *UHRF1, MKI67, TRNP1, MLH1, PTTG1* | *40,00* | *614,00* | *18082,00* | | *3,68* | | *1,00* | | *0,76* | *43,90* |  |  |
| GOTERM_BP_DIRECT | GO:0007126~meiotic nuclear division | | 2,00 | | 0,03 | 0,05 | *1,30* | | MKI67, MLH1 | 40,00 | 24,00 | 18082,00 | | 37,67 | | 1,00 | | 0,79 | 49,94 |  |  |
| GOTERM_BP_DIRECT | GO:0045087~innate immune response | | 4,00 | | 0,06 | 0,05 | *1,26* | | HMGB2, C1RB, C4B, PGLYRP1 | 40,00 | 400,00 | 18082,00 | | 4,52 | | 1,00 | | 0,78 | 52,76 |  |  |
| GOTERM_BP_DIRECT | GO:0006974~cellular response to DNA damage stimulus | | 4,00 | | 0,06 | 0,06 | *1,21* | | UHRF1, MLH1, PTTG1, TOP2A | 40,00 | 420,00 | 18082,00 | | 4,31 | | 1,00 | | 0,79 | 57,11 |  |  |
| GOTERM_BP_DIRECT | GO:0003341~cilium movement | | 2,00 | | 0,03 | 0,07 | *1,14* | | DNAH10, HYDIN | 40,00 | 35,00 | 18082,00 | | 25,83 | | 1,00 | | 0,82 | 63,55 |  |  |
|  |  | |  | |  |  |  | |  |  |  |  | |  | |  | |  |  |  |  |
|  |  | |  | |  |  |  | |  |  |  |  | |  | |  | |  |  |  |  |
| **KEGG Pathway analysis** | | | | | | | | | | | | | | | | | | | | | |
| **Category** | **Term** | **Count** | | **%** | | **PValue** | **LOG(Pvalue)** | **Genes** | | **List Total** | **Pop Hits** | | **Pop Total** | | **Fold Enrichment** | **Bonferroni** | | **Benjamini** | **FDR** | |  |
| KEGG_PATHWAY | mmu05016:Huntington's disease | 3,00 | | 0,04 | | 0,03 | 1,59 | DNAH10, DNAH12, DNAH3 | | 11,00 | 198,00 | | 7720,00 | | 10,63 | 0,52 | | 0,52 | 19,59 | |  |
| KEGG_PATHWAY | mmu05144:Malaria | 2,00 | | 0,03 | | 0,06 | 1,22 | HBA-A2, THBS4 | | 11,00 | 48,00 | | 7720,00 | | 29,24 | 0,83 | | 0,58 | 40,67 | |  |
|  |  |  | |  | |  |  |  | |  |  | |  | |  |  | |  |  | |  |
|  |  |  | |  | |  |  |  | |  |  | |  | |  |  | |  |  | |  |
| **Molecular Function analysis** | | | | | | | | | | | | | | | | | | | | | |
| **Category** | **Term** | **Count** | | **%** | | **PValue** | **LOG(Pvalue)** | **Genes** | | **List Total** | **Pop Hits** | | **Pop Total** | | **Fold Enrichment** | **Bonferroni** | **Benjamini** | | **FDR** | |  |
| GOTERM_MF_DIRECT | GO:0003777~microtubule motor activity | 4,00 | | 0,06 | | 0,00 | 3,22 | DNAH10, DNAH12, KIF17, DNAH3 | | 39,00 | 76,00 | | 17446,00 | | 23,54 | 0,08 | 0,08 | | 0,70 | |  |
| GOTERM_MF_DIRECT | GO:0008301~DNA binding, bending | 2,00 | | 0,03 | | 0,03 | 1,46 | HMGB2, TOP2A | | 39,00 | 16,00 | | 17446,00 | | 55,92 | 0,99 | 0,91 | | 33,65 | |  |
| GOTERM_MF_DIRECT | GO:0001968~fibronectin binding | 2,00 | | 0,03 | | 0,06 | 1,21 | THBS4, IGFBP5 | | 39,00 | 29,00 | | 17446,00 | | 30,85 | 1,00 | 0,94 | | 52,47 | |  |
| GOTERM_MF_DIRECT | GO:0004866~endopeptidase inhibitor activity | 2,00 | | 0,03 | | 0,07 | 1,18 | C4B, PCSK1N | | 39,00 | 31,00 | | 17446,00 | | 28,86 | 1,00 | 0,90 | | 54,85 | |  |
| GOTERM_MF_DIRECT | GO:0016887~ATPase activity | 3 | | 0,04404 | | 0,07025 | 1,15335 | DNAH10, DNAH12, MLH1 | | 39 | 200 | | 17446 | | 6,71 | 0,99995 | 0,86410 | | 57,52166 | |  |
| GOTERM_MF_DIRECT | GO:0008022~protein C-terminus binding | 3 | | 0,04404 | | 0,07581 | 1,12026 | MKI67, CENPF, TOP2A | | 39 | 209 | | 17446 | | 6,42105263 | 0,99998 | 0,83473 | | 60,41461 | |  |
