# Supplementary material for: The longevity-associated variant of BPIFB4 improves a CXCR4-mediated striatum–microglia crosstalk preventing disease progression in a mouse model of Huntington’s disease
Source: Cell Death Dis. 2020 Jul 18;11(7):546. doi: 10.1038/s41419-020-02754-w (PMC7368858; doi:10.1038/s41419-020-02754-w)
Supplement: Supplementary file 7 — Supplementary information 7 [file 41419_2020_2754_MOESM7_ESM.docx]

| **GENE ONTOLOGY WT-BPIFB4 VS LAV-BPIFB4** | | | | | | | | | | | | | | | |  |
| --- | --- | --- | --- | --- | --- | --- | --- | --- | --- | --- | --- | --- | --- | --- | --- | --- |
|  |  |  |  |  |  |  |  |  |  |  |  |  |  | | | |
|  |  |  |  |  |  |  |  |  |  |  |  |  |  | | | |
| **Biological Process Analysis** | | | | | | | | | | | | | | | |  |
| **Category** | **Term** | **Count** | **%** | **PValue** | **LOG(PValue)** | **Genes** | **List Total** | **Pop Hits** | **Pop Total** | **Fold Enrichment** | **Bonferroni** | **Benjamini** | **FDR** | |  |  |
| GOTERM_BP_DIRECT | GO:0007626~locomotory behavior | 5 | 0,10951 | 1,8999E-05 | 4,72126 | ADORA2A, DRD2, CHRNA4, GPR88, RASD2 | 28 | 108 | 18082 | 29,89749 | 0,00672 | 0,00672 | 0,02604 | |  |  |
| GOTERM_BP_DIRECT | GO:0001963~synaptic transmission, dopaminergic | 3 | 0,06570 | 0,00025 | 3,59456 | ADORA2A, DRD2, RASD2 | 28 | 16 | 18082 | 121,08482 | 0,08635 | 0,04415 | 0,34805 | |  |  |
| GOTERM_BP_DIRECT | GO:0001975~response to amphetamine | 3 | 0,06570 | 0,00117 | 2,93197 | PPP1R1B, ADORA2A, DRD2 | 28 | 34 | 18082 | 56,98109 | 0,33995 | 0,12932 | 1,59112 | |  |  |
| GOTERM_BP_DIRECT | GO:0042493~response to drug | 5 | 0,10951 | 0,00151 | 2,81968 | LPL, FOS, ADORA2A, DRD2, JUNB | 28 | 339 | 18082 | 9,52486 | 0,41615 | 0,12587 | 2,05609 | |  |  |
| GOTERM_BP_DIRECT | GO:0035094~response to nicotine | 3 | 0,06570 | 0,00204 | 2,68969 | DRD2, CNR1, CHRNA4 | 28 | 45 | 18082 | 43,05238 | 0,51619 | 0,13516 | 2,76425 | |  |  |
| GOTERM_BP_DIRECT | GO:0019233~sensory perception of pain | 3 | 0,06570 | 0,00586 | 2,23181 | CNR1, CHRNA4, MME | 28 | 77 | 18082 | 25,16048 | 0,87604 | 0,29388 | 7,74419 | |  |  |
| GOTERM_BP_DIRECT | GO:0034097~response to cytokine | 3 | 0,06570 | 0,00647 | 2,18914 | FOS, SERPINA3N, JUNB | 28 | 81 | 18082 | 23,91799 | 0,90015 | 0,28047 | 8,51135 | |  |  |
| GOTERM_BP_DIRECT | GO:0033602~negative regulation of dopamine secretion | 2 | 0,04380 | 0,00744 | 2,12816 | DRD2, CNR1 | 28 | 5 | 18082 | 258,31429 | 0,92954 | 0,28222 | 9,73441 | |  |  |
| GOTERM_BP_DIRECT | GO:0007165~signal transduction | 7 | 0,15331 | 0,00924 | 2,03412 | PPP1R1B, ADORA2A, DRD2, CNR1, CHRNA4, GPR88, RASD2 | 28 | 1255 | 18082 | 3,60199 | 0,96301 | 0,30673 | 11,95221 | |  |  |
| GOTERM_BP_DIRECT | GO:0034765~regulation of ion transmembrane transport | 3 | 0,06570 | 0,01719 | 1,76473 | KCNC2, SCN4B, KCNG1 | 28 | 135 | 18082 | 14,35079 | 0,99788 | 0,45965 | 21,15185 | |  |  |
| GOTERM_BP_DIRECT | GO:0060080~inhibitory postsynaptic potential | 2 | 0,04380 | 0,01924 | 1,71568 | ADORA2A, CHRNA4 | 28 | 13 | 18082 | 99,35165 | 0,99899 | 0,46588 | 23,38176 | |  |  |
| GOTERM_BP_DIRECT | GO:0032228~regulation of synaptic transmission, GABAergic | 2 | 0,04380 | 0,01924 | 1,71568 | DRD2, CNR1 | 28 | 13 | 18082 | 99,35165 | 0,99899 | 0,46588 | 23,38176 | |  |  |
| GOTERM_BP_DIRECT | GO:0035815~positive regulation of renal sodium excretion | 2 | 0,04380 | 0,01924 | 1,71568 | ADORA2A, DRD2 | 28 | 13 | 18082 | 99,35165 | 0,99899 | 0,46588 | 23,38176 | |  |  |
| GOTERM_BP_DIRECT | GO:0035810~positive regulation of urine volume | 2 | 0,04380 | 0,02071 | 1,68381 | ADORA2A, DRD2 | 28 | 14 | 18082 | 92,25510 | 0,99941 | 0,46158 | 24,93609 | |  |  |
| GOTERM_BP_DIRECT | GO:0060134~prepulse inhibition | 2 | 0,04380 | 0,02217 | 1,65416 | ADORA2A, DRD2 | 28 | 15 | 18082 | 86,10476 | 0,99965 | 0,45791 | 26,45897 | |  |  |
| GOTERM_BP_DIRECT | GO:0014059~regulation of dopamine secretion | 2 | 0,04380 | 0,02217 | 1,65416 | DRD2, CHRNA4 | 28 | 15 | 18082 | 86,10476 | 0,99965 | 0,45791 | 26,45897 | |  |  |
| GOTERM_BP_DIRECT | GO:0001508~action potential | 2 | 0,04380 | 0,02801 | 1,55274 | KCNC2, CHRNA4 | 28 | 19 | 18082 | 67,97744 | 0,99996 | 0,51339 | 32,24850 | |  |  |
| GOTERM_BP_DIRECT | GO:0051899~membrane depolarization | 2 | 0,04380 | 0,03381 | 1,47101 | ADORA2A, CHRNA4 | 28 | 23 | 18082 | 56,15528 | 1,00000 | 0,55687 | 37,58338 | |  |  |
| GOTERM_BP_DIRECT | GO:0032496~response to lipopolysaccharide | 3 | 0,06570 | 0,03468 | 1,45996 | FOS, CNR1, JUNB | 28 | 197 | 18082 | 9,83430 | 1,00000 | 0,54299 | 38,35024 | |  |  |
| GOTERM_BP_DIRECT | GO:0009416~response to light stimulus | 2 | 0,04380 | 0,03669 | 1,43542 | FOS, DRD2 | 28 | 25 | 18082 | 51,66286 | 1,00000 | 0,54189 | 40,09157 | |  |  |
| GOTERM_BP_DIRECT | GO:0043278~response to morphine | 2 | 0,04380 | 0,03813 | 1,41870 | DRD2, CNR1 | 28 | 26 | 18082 | 49,67582 | 1,00000 | 0,53549 | 41,30771 | |  |  |
| GOTERM_BP_DIRECT | GO:0007271~synaptic transmission, cholinergic | 2 | 0,04380 | 0,04387 | 1,35779 | ADORA2A, CHRNA4 | 28 | 30 | 18082 | 43,05238 | 1,00000 | 0,56754 | 45,93098 | |  |  |
| GOTERM_BP_DIRECT | GO:0007188~adenylate cyclase-modulating G-protein coupled receptor signaling pathway | 2 | 0,04380 | 0,04958 | 1,30468 | DRD2, CNR1 | 28 | 34 | 18082 | 37,98739 | 1,00000 | 0,59450 | 50,19097 | |  |  |
| GOTERM_BP_DIRECT | GO:0045776~negative regulation of blood pressure | 2 | 0,04380 | 0,05100 | 1,29240 | DRD2, CNR1 | 28 | 35 | 18082 | 36,90204 | 1,00000 | 0,58727 | 51,20259 | |  |  |
| GOTERM_BP_DIRECT | GO:0006811~ion transport | 4 | 0,08760 | 0,05522 | 1,25789 | KCNC2, CHRNA4, SCN4B, KCNG1 | 28 | 584 | 18082 | 4,42319 | 1,00000 | 0,60013 | 54,09260 | |  |  |
| GOTERM_BP_DIRECT | GO:0042220~response to cocaine | 2 | 0,04380 | 0,05526 | 1,25761 | DRD2, CNR1 | 28 | 38 | 18082 | 33,98872 | 1,00000 | 0,58411 | 54,11616 | |  |  |
| GOTERM_BP_DIRECT | GO:0007154~cell communication | 2 | 0,04380 | 0,05667 | 1,24664 | FREM2, KREMEN1 | 28 | 39 | 18082 | 33,11722 | 1,00000 | 0,57808 | 55,04827 | |  |  |
| GOTERM_BP_DIRECT | GO:0009409~response to cold | 2 | 0,04380 | 0,05808 | 1,23596 | LPL, FOS | 28 | 40 | 18082 | 32,28929 | 1,00000 | 0,57245 | 55,96149 | |  |  |
| GOTERM_BP_DIRECT | GO:0032870~cellular response to hormone stimulus | 2 | 0,04380 | 0,06791 | 1,16809 | FOS, JUNB | 28 | 47 | 18082 | 27,48024 | 1,00000 | 0,61717 | 61,85636 | |  |  |
| GOTERM_BP_DIRECT | GO:0051591~response to cAMP | 2 | 0,04380 | 0,07348 | 1,13385 | FOS, JUNB | 28 | 51 | 18082 | 25,32493 | 1,00000 | 0,63337 | 64,86434 | |  |  |
| GOTERM_BP_DIRECT | GO:0071277~cellular response to calcium ion | 2 | 0,04380 | 0,07625 | 1,11777 | FOS, JUNB | 28 | 53 | 18082 | 24,36927 | 1,00000 | 0,63417 | 66,27841 | |  |  |
| GOTERM_BP_DIRECT | GO:0035914~skeletal muscle cell differentiation | 2 | 0,04380 | 0,07901 | 1,10230 | FOS, NR4A1 | 28 | 55 | 18082 | 23,48312 | 1,00000 | 0,63490 | 67,63571 | |  |  |
| GOTERM_BP_DIRECT | GO:0008542~visual learning | 2 | 0,04380 | 0,08999 | 1,04579 | PPP1R1B, DRD2 | 28 | 63 | 18082 | 20,50113 | 1,00000 | 0,67239 | 72,54140 | |  |  |
| GOTERM_BP_DIRECT | GO:0043154~negative regulation of cysteine-type endopeptidase activity involved in apoptotic process | 2 | 0,04380 | 0,09544 | 1,02029 | ADORA2A, NR4A1 | 28 | 67 | 18082 | 19,27719 | 1,00000 | 0,68293 | 74,70861 | |  |  |
|  |  |  |  |  |  |  |  |  |  |  |  |  |  | |  |  |
|  |  |  |  |  |  |  |  |  |  |  |  |  |  | |  |  |
| **KEGG Pathway analysis** | | | | | | | | | | | | | |  |  |  |
| **Category** | **Term** | **Count** | **%** | **PValue** | **LOG(PValue)** | **Genes** | **List Total** | **Pop Hits** | **Pop Total** | **Fold Enrichment** | **Bonferroni** | **Benjamini** | **FDR** |  |  |  |
| KEGG_PATHWAY | mmu04024:cAMP signaling pathway | 4 | 0,08760 | 0,00304 | 2,51754 | FOS, PPP1R1B, ADORA2A, DRD2 | 13 | 197 | 7720 | 12,05779 | 0,15405 | 0,15405 | 2,94745 |  |  |  |
| KEGG_PATHWAY | mmu04080:Neuroactive ligand-receptor interaction | 4 | 0,08760 | 0,00855 | 2,06814 | ADORA2A, DRD2, CNR1, CHRNA4 | 13 | 285 | 7720 | 8,33468 | 0,37635 | 0,21028 | 8,09715 |  |  |  |
| KEGG_PATHWAY | mmu04728:Dopaminergic synapse | 3 | 0,06570 | 0,01761 | 1,75417 | FOS, PPP1R1B, DRD2 | 13 | 134 | 7720 | 13,29506 | 0,62369 | 0,27803 | 16,03574 |  |  |  |
| KEGG_PATHWAY | mmu05034:Alcoholism | 3 | 0,06570 | 0,03784 | 1,42204 | PPP1R1B, ADORA2A, DRD2 | 13 | 202 | 7720 | 8,81950 | 0,88017 | 0,41164 | 31,57420 |  |  |  |
| KEGG_PATHWAY | mmu04015:Rap1 signaling pathway | 3 | 0,06570 | 0,04205 | 1,37628 | ADORA2A, DRD2, CNR1 | 13 | 214 | 7720 | 8,32495 | 0,90582 | 0,37656 | 34,45910 |  |  |  |
| KEGG_PATHWAY | mmu05030:Cocaine addiction | 2 | 0,04380 | 0,07361 | 1,13305 | PPP1R1B, DRD2 | 13 | 49 | 7720 | 24,23862 | 0,98509 | 0,50387 | 52,86147 |  |  |  |
| KEGG_PATHWAY | mmu05031:Amphetamine addiction | 2 | 0,04380 | 0,09938 | 1,00269 | FOS, PPP1R1B | 13 | 67 | 7720 | 17,72675 | 0,99684 | 0,56064 | 64,28364 |  |  |  |
|  |  |  |  |  |  |  |  |  |  |  |  |  |  |  |  |  |
|  |  |  |  |  |  |  |  |  |  |  |  |  |  |  |  |  |
| **Molecular Function analysis** | | | | | | | | | | | | | |  |  |  |
| **Category** | **Term** | **Count** | **%** | **PValue** | **LOG(PValue)** | **Genes** | **List Total** | **Pop Hits** | **Pop Total** | **Fold Enrichment** | **Bonferroni** | **Benjamini** | **FDR** | | | |
| GOTERM_MF_DIRECT | GO:0046982~protein heterodimerization activity | 6 | 0,13141 | 7,10E-04 | 3,14899 | FOS, ADORA2A, DRD2, CHRNA4, NR4A1, NRN1 | 26 | 514 | 17446 | 7,83268 | 0,07708 | 0,07708 | 0,80274 | | | |
| GOTERM_MF_DIRECT | GO:0008144~drug binding | 3 | 0,06570 | 0,01113 | 1,95356 | DRD2, CNR1, CHRNA4 | 26 | 112 | 17446 | 17,97321 | 0,71764 | 0,46863 | 11,93245 | | | |
| GOTERM_MF_DIRECT | GO:0044325~ion channel binding | 3 | 0,06570 | 0,01269 | 1,89640 | KCNC2, SCN4B, ACTN2 | 26 | 120 | 17446 | 16,77500 | 0,76392 | 0,38196 | 13,50241 | | | |
| GOTERM_MF_DIRECT | GO:0005244~voltage-gated ion channel activity | 3 | 0,06570 | 0,01565 | 1,80548 | KCNC2, SCN4B, KCNG1 | 26 | 134 | 17446 | 15,02239 | 0,83178 | 0,35957 | 16,39813 | | | |
| GOTERM_MF_DIRECT | GO:0005216~ion channel activity | 3 | 0,06570 | 0,02445 | 1,61170 | KCNC2, CHRNA4, KCNG1 | 26 | 170 | 17446 | 11,84118 | 0,93903 | 0,42848 | 24,50289 | | | |
| GOTERM_MF_DIRECT | GO:0042803~protein homodimerization activity | 5 | 0,10951 | 0,02555 | 1,59259 | DRD2, NR4A1, ACTN2, NRN1, CRYM | 26 | 798 | 17446 | 4,20426 | 0,94633 | 0,38582 | 25,46398 | | | |
| GOTERM_MF_DIRECT | GO:0005251~delayed rectifier potassium channel activity | 2 | 0,04380 | 0,04489 | 1,34784 | KCNC2, KCNG1 | 26 | 32 | 17446 | 41,93750 | 0,99443 | 0,52357 | 40,63690 | | | |
| GOTERM_MF_DIRECT | GO:0001077~transcriptional activator activity, RNA polymerase II core promoter proximal region sequence-specific binding | 3 | 0,06570 | 0,05669 | 1,24652 | FOS, NR4A1, JUNB | 26 | 270 | 17446 | 7,45556 | 0,99863 | 0,56145 | 48,44897 | | | |
| GOTERM_MF_DIRECT | GO:0004871~signal transducer activity | 4 | 0,08760 | 0,06401 | 1,19375 | ADORA2A, DRD2, CNR1, GPR88 | 26 | 648 | 17446 | 4,14198 | 0,99943 | 0,56420 | 52,81515 | | | |
| GOTERM_MF_DIRECT | GO:0003779~actin binding | 3 | 0,06570 | 0,08385 | 1,07649 | INF2, ACTN2, EPS8L1 | 26 | 338 | 17446 | 5,95562 | 0,99995 | 0,62828 | 63,00440 | | | |
| GOTERM_MF_DIRECT | GO:0008134~transcription factor binding | 3 | 0,06570 | 0,08556 | 1,06775 | FOS, NR4A1, JUNB | 26 | 342 | 17446 | 5,88596 | 0,99996 | 0,60100 | 63,77883 | | | |
| GOTERM_MF_DIRECT | GO:0005249~voltage-gated potassium channel activity | 2 | 0,04380 | 0,09569 | 1,01914 | KCNC2, KCNG1 | 26 | 70 | 17446 | 19,17143 | 0,99999 | 0,61215 | 68,08271 | | | |
