# Supplementary material for: The longevity-associated variant of BPIFB4 improves a CXCR4-mediated striatum–microglia crosstalk preventing disease progression in a mouse model of Huntington’s disease
Source: Cell Death Dis. 2020 Jul 18;11(7):546. doi: 10.1038/s41419-020-02754-w (PMC7368858; doi:10.1038/s41419-020-02754-w)
Supplement: Supplementary file 8 — Supplementary information 8 [file 41419_2020_2754_MOESM8_ESM.docx]

| **GENE ONTOLOGY WT-BPIFB4 VS EMPTY VECTOR** | | | | | | | | | | | | | | | | | | | | | | | | | | |  |
| --- | --- | --- | --- | --- | --- | --- | --- | --- | --- | --- | --- | --- | --- | --- | --- | --- | --- | --- | --- | --- | --- | --- | --- | --- | --- | --- | --- |
|  |  | |  | |  | |  | |  | |  | |  | |  | |  | |  | |  | |  | |  | | |
|  |  | |  | |  | |  | |  | |  | |  | |  | |  | |  | |  | |  | |  | | |
| **Biological Process Analysis** | | | | | | | | | | | | | | | | | | | | | | | | | | |  |
| **Category** | **Term** | **Count** | | **%** | | **PValue** | | **LOG(PValue)** | | **Genes** | | **List Total** | | **Pop Hits** | | **Pop Total** | | **Fold Enrichment** | | **Bonferroni** | | **Benjamini** | | **FDR** | |  |  |
| GOTERM_BP_DIRECT | GO:0007275~multicellular organism development | 9 | | 0,11180 | | 0,01084 | | 1,96508 | | SHOX2, SATB2, HYDIN, NNAT, SEMA3E, PSME4, SEMA3A, UTP14B, QK | | 55 | | 1029 | | 18082 | | 2,87548 | | 0,97071 | | 0,97071 | | 13,69798 | |  |  |
| GOTERM_BP_DIRECT | GO:0050919~negative chemotaxis | 2 | | 0,02484 | | 0,07487 | | 1,12572 | | SEMA3E, SEMA3A | | 55 | | 26 | | 18082 | | 25,28951 | | 1,00000 | | 1,00000 | | 65,07817 | |  |  |
| GOTERM_BP_DIRECT | GO:0048843~negative regulation of axon extension involved in axon guidance | 2 | | 0,02484 | | 0,07487 | | 1,12572 | | SEMA3E, SEMA3A | | 55 | | 26 | | 18082 | | 25,28951 | | 1,00000 | | 1,00000 | | 65,07817 | |  |  |
| GOTERM_BP_DIRECT | GO:0071310~cellular response to organic substance | 2 | | 0,02484 | | 0,09406 | | 1,02658 | | SATB2, NR4A1 | | 55 | | 33 | | 18082 | | 19,92507 | | 1,00000 | | 0,99998 | | 73,69902 | |  |  |
| GOTERM_BP_DIRECT | GO:0071526~semaphorin-plexin signaling pathway | 2 | | 0,02484 | | 0,09677 | | 1,01424 | | SEMA3E, SEMA3A | | 55 | | 34 | | 18082 | | 19,33904 | | 1,00000 | | 0,99974 | | 74,74317 | |  |  |
| GOTERM_BP_DIRECT | GO:0003341~cilium movement | 2 | | 0,02484 | | 0,09948 | | 1,00228 | | DNAH10, HYDIN | | 55 | | 35 | | 18082 | | 18,78649 | | 1,00000 | | 0,99887 | | 75,74593 | |  |  |
|  |  |  | |  | |  | |  | |  | |  | |  | |  | |  | |  | |  | |  | |  |  |
|  |  |  | |  | |  | |  | |  | |  | |  | |  | |  | |  | |  | |  | |  |  |
| **KEGG Pathway analysis** | | | | | | | | | | | | | | | | | | | | | | | | | | |  |
| **Category** | **Term** | | **Count** | | **%** | | **PValue** | | **LOG(PValue)** | | **Genes** | | **List Total** | | **Pop Hits** | | **Pop Total** | | **Fold Enrichment** | | **Bonferroni** | | **Benjamini** | | **FDR** | | |
| KEGG_PATHWAY | mmu05016:Huntington's disease | | 6 | | 0,07453 | | 0,00019 | | 3,71068 | | DNAH10, DNAH12, UQCRH, DNAH3, COX5B, NDUFA12 | | 23 | | 198 | | 7720 | | 10,17128 | | 0,00602 | | 0,00602 | | 0,16712 | | |
| KEGG_PATHWAY | mmu05010:Alzheimer's disease | | 4 | | 0,04969 | | 0,01324 | | 1,87800 | | UQCRH, IDE, COX5B, NDUFA12 | | 23 | | 177 | | 7720 | | 7,58536 | | 0,33853 | | 0,18669 | | 10,82161 | | |
| KEGG_PATHWAY | mmu00190:Oxidative phosphorylation | | 3 | | 0,03727 | | 0,05879 | | 1,23066 | | UQCRH, COX5B, NDUFA12 | | 23 | | 139 | | 7720 | | 7,24429 | | 0,84717 | | 0,46535 | | 40,58067 | | |
| KEGG_PATHWAY | mmu03010:Ribosome | | 3 | | 0,03727 | | 0,06335 | | 1,19825 | | RPS27, RPS29, RPL28 | | 23 | | 145 | | 7720 | | 6,94453 | | 0,86851 | | 0,39782 | | 43,00666 | | |
| KEGG_PATHWAY | mmu05012:Parkinson's disease | | 3 | | 0,03727 | | 0,06645 | | 1,17748 | | UQCRH, COX5B, NDUFA12 | | 23 | | 149 | | 7720 | | 6,75810 | | 0,88137 | | 0,34711 | | 44,60867 | | |
| KEGG_PATHWAY | mmu04932:Non-alcoholic fatty liver disease (NAFLD) | | 3 | | 0,03727 | | 0,07281 | | 1,13779 | | UQCRH, COX5B, NDUFA12 | | 23 | | 157 | | 7720 | | 6,41374 | | 0,90402 | | 0,32335 | | 47,76745 | | |
|  |  | |  | |  | |  | |  | |  | |  | |  | |  | |  | |  | |  | |  | | |
|  |  | |  | |  | |  | |  | |  | |  | |  | |  | |  | |  | |  | |  | | |
| **Molecular Function analysis** | | | | | | | | | | | | | | | | | | | | | | | | | | |  |
| **Category** | **Term** | | **Count** | | **%** | | **PValue** | | **LOG(PValue)** | | **Genes** | | **List Total** | | **Pop Hits** | | **Pop Total** | | **Fold Enrichment** | | **Bonferroni** | | **Benjamini** | | **FDR** | | |
| GOTERM_MF_DIRECT | GO:0005518~collagen binding | | 3 | | 0,03727 | | 0,01058 | | 1,97561 | | VWF, THBS4, RELL2 | | 47 | | 59 | | 17446 | | 18,87414 | | 0,72382 | | 0,72382 | | 11,50769 | | |
| GOTERM_MF_DIRECT | GO:0003777~microtubule motor activity | | 3 | | 0,03727 | | 0,01713 | | 1,76630 | | DNAH10, DNAH12, DNAH3 | | 47 | | 76 | | 17446 | | 14,65230 | | 0,87636 | | 0,64838 | | 18,01343 | | |
| GOTERM_MF_DIRECT | GO:0038191~neuropilin binding | | 2 | | 0,02484 | | 0,02090 | | 1,67977 | | SEMA3E, SEMA3A | | 47 | | 8 | | 17446 | | 92,79787 | | 0,92240 | | 0,57347 | | 21,56295 | | |
| GOTERM_MF_DIRECT | GO:0030215~semaphorin receptor binding | | 2 | | 0,02484 | | 0,05397 | | 1,26789 | | SEMA3E, SEMA3A | | 47 | | 21 | | 17446 | | 35,35157 | | 0,99878 | | 0,81328 | | 47,15366 | | |
| GOTERM_MF_DIRECT | GO:0045499~chemorepellent activity | | 2 | | 0,02484 | | 0,06885 | | 1,16208 | | SEMA3E, SEMA3A | | 47 | | 27 | | 17446 | | 27,49567 | | 0,99982 | | 0,82207 | | 55,96328 | | |
| GOTERM_MF_DIRECT | GO:0016887~ATPase activity | | 3 | | 0,03727 | | 0,09755 | | 1,01077 | | DNAH10, DNAH12, IDE | | 47 | | 200 | | 17446 | | 5,56787 | | 1,00000 | | 0,87381 | | 69,27320 | | |
