# Supplementary material for: The longevity-associated variant of BPIFB4 improves a CXCR4-mediated striatum–microglia crosstalk preventing disease progression in a mouse model of Huntington’s disease
Source: Cell Death Dis. 2020 Jul 18;11(7):546. doi: 10.1038/s41419-020-02754-w (PMC7368858; doi:10.1038/s41419-020-02754-w)
Supplement: Supplementary file 9 — Supplementary information 9 [file 41419_2020_2754_MOESM9_ESM.docx]

**Supplementary materials’ legend**

**Supplementary Figures’ legend**

- **Supplementary figure 1. Overexpression of WT-BPIFB4 doesn’t ameliorate motor function of R6/2 mice.** Horizontal Ladder Task and Rotarod analyses of motor performance in treated and untreated R6/2 mice and age- and gender-matched wild-type (B6) littermates. Each data point represents the average performance ± SD of 6–9 mice per group. Conversely to AAV-LAV-BPIFB4 treatment, AAV-WT-BPIFB4 does not improve motor function.
- Supplementary figure 2. Principal Component Analyses and Euclidean Distance Analysis
- **Supplementary Figure 2. Principal Component Analyses and Euclidean Distance Analysis:** Graphics show the results of **A)** Principal Component Analysis and **B)** Euclidean Distance Analysis of filtered dataset. 4 outliers were excluded: 2 AAV-GFP and 2 AAV-LAV-BPIFB4 injected mice.

**Supplementary Tables**

- Supplementary Table 1. Differential expression (RNAseq analysis) in Striatum of mice infected with Empty Vector compared to LAV-BPIFB4 (FDR<=5).

- Supplementary Table 2. Differential expression (RNAseq analysis) in Striatum of mice infected with WT-BPIFB4 compared to LAV-BPIFB4 (FDR<=5).

- Supplementary Table 3. Differential expression (RNAseq analysis) of Striatum of mice infected with WT-BPIFB4 compared to Empty Vector (FDR<=5).

- Supplementary Table 4. Striatum Gene Ontology analysis: Empty Vector Vs LAV-BPIFB4.

- Supplementary Table 5. Striatum Gene Ontology analysis: WT-BPIFB4 Vs LAV-BPIFB4.

- Supplementary Table 6. Striatum Gene Ontology analysis: WT-BPIFB4 Vs Empty Vector.

.
